# Sexual behavior and cardiovascular diseases: univariable and multivariable Mendelian randomization

**DOI:** 10.3389/fcvm.2023.1250404

**Published:** 2023-12-05

**Authors:** Kejing Zhu, Zhongliang Lin, Qinyu Luo, Zhaoying Jiang, Renke He, Haiyan Wu, Jiaen Yu, Xueying Liu, Jianzhong Sheng, Hong Zhu, Hefeng Huang

**Affiliations:** ^1^ Department of Obstetrics and Gynecology, The Fourth Affiliated Hospital, International Institutes of Medicine, Zhejiang University School of Medicine, Yiwu, China; ^2^ Key Laboratory of Reproductive Genetics (Ministry of Education), Department of Reproductive Endocrinology, Women’s Hospital, Zhejiang University School of Medicine, Hangzhou, China; ^3^ Research Units of Embryo Original Diseases, Chinese Academy of Medical Sciences (No. 2019RU056), Shanghai, China; ^4^ Obstetrics and Gynecology Hospital, Institute of Reproduction and Development, Fudan University, Shanghai, China; ^5^ Shanghai Key Laboratory of Reproduction and Development, Shanghai, China; ^6^ State Key Laboratory of Cardiology, Shanghai, China

**Keywords:** cardiovascular diseases, sexual behaviors, Mendelian randomization, causal relationship, statistic method

## Abstract

**Background:**

To assess the relationship of genetically predicted sexual behavior (age at first sex (AFS) and the number of sexual partners (NSP)) on cardiovascular diseases (CVDs).

**Methods and results:**

We performed two-sample Mendelian randomization (MR) with publicly available datasets from the UK Biobank and FinnGen Study, and analyzed genome-wide association results for sexual behaviors and twelve types of CVDs. The univariable MR method provided a total effect of AFS and NSP on CVDs, and showed evidence that early AFS rather than NSP was associated with CVDs, including angina pectoris (AP), atrial fibrillation and flutter (AFF), coronary atherosclerosis (CAS), deep vein thrombosis of the lower extremity (DVT-LE), heart failure (HF), hypertension (HTN), ischaemic stroke (IS), and myocardial infarction (MI). Given sex as a social determinant of CVD risk, we used gender-stratified SNPs to investigate gender differences in the development of CVDs. These results showed a stronger causal relationship of AFS on CVDs in females than in males. Further multivariable MR analyses indicated a direct effect after accounting for insomnia, number of days of vigorous physical activity 10 + minutes (VPA 10 + min), and time spent watching television (TV). Two-step MR demonstrated these three risk factors act as a mediator in AFS associated AP/HTN/HF.

**Conclusions:**

We provide evidence that early AFS increased the risk of CVDs. These associations may be partly caused by VPA 10 + min, insomnia, and the time spent on TV. The causality of AFS on CVDs in females was stronger than in males. Conversely, genetically predicted NSP was not associated with CVDs.

## Introduction

Cardiovascular disease (CVD) is now the leading cause of mortality and morbidity among noncommunicable diseases, and has become the largest contributor to disease burden in the world. According to the Global Burden of Disease Study 2019, it is estimated that a total of approximately 523 million people suffer from cardiovascular disease (1, 2). Over the last 20 years, despite the general improvement in the cardiovascular health of middle-aged and older adults individuals, it is worrisome that younger adults have tended to develop CVD because of the progressive unhealthy cardiovascular risk profile (3, 4). A large body of data suggests that the majority of cardiovascular disease cases are associated with several common and modifiable risk factors, including smoking, regular alcohol consumption, lack of physical activity, etc. (2).

Sexual activity is a key aspect of healthy life. Several observational studies indicate a significant correlation between sexual activity and acute cardiac events (5, 6). Age at first sex (AFS) and the number of sexual partners (NSP) are determinants of sexual activity. The two are linked as those who were younger when they first had sex are may have more sexual partners. Furthermore, these sexual behaviors are associated with psychiatric disorders (6, 7), sexually transmitted diseases (8), oral cancer (9), prostate cancer (10), and cervical cancer (11). An observational study showed a negative association between AFS and hypertension (12). However, traditional epidemiology, with its potential confounders and various biases, makes it difficult to determine whether sex behaviors are causal or merely shared pleiotropic factors for CVDs risk.

With the rapid development of genome-wide association studies (GWAS), the availability of a large amount of aggregated data provides an opportunity for the widespread application of Mendelian randomization (MR) analysis. MR is an alternative strategy to investigate potential causal inferences between exposure and outcome using phenotypically closely related single nucleotide polymorphisms (SNPs) as instrumental variables (IVs). Since SNPs are randomly assigned at the time of conception, MR can be considered as a natural randomized controlled trial, thus avoiding the effects of measurement error, confounding factors, and reverse causality in observational studies (13).

A genome-wide screen identifies early AFS as a causal risk factor for coronary artery disease (14).In addition, a previous MR study has found that lower AFS was significantly associated with an increased risk of intracerebral hemorrhage or small vessel stroke (15). Nevertheless, a previous MR study has found that the causal effects of the two sexual behaviors on several types of CVDs. Notably, gender is considered a social determinant of CVD (16), which has been overlooked in most MR studies. Additionally, the rising prevalence of CVD is based partly on less physical activity and more non-exercise behaviours, including insomnia, less days of vigorous physical activity over 10 min (VPA 10 + min), and more time spent watching television (TV) (5, 17).

In this study, we performed a two-sample MR analysis to further fully elucidate whether sexual behavior has a causal effect on twelve CVDs, including angina pectoris (AP), atrial fibrillation and flutter (AFF), coronary atherosclerosis (CAS), aortic dissection (AD), deep vein thrombosis of the lower extremity (DVT-LE), heart failure (HF), hypertension (HTN), intracerebral hemorrhage (ICH), ischaemic stroke (IS), myocardial infarction (MI), pulmonary embolism (PE), and subarachnoid hemorrhage (SAH). An array of MR designs, including univariable MR (UVMR), multivariate MR (MVMR), and two-step MR were used to explore the direct and total effects of sexual behavior on CVDs and to illustrate the respective roles of TV, VPA 10 + min, and insomnia. Assessing these health-related risk factors for CVD may go a long way in optimizing disease prevention internationally at the clinical and public health levels.

## Study design

In this study, we performed two-sample MR with publicly available datasets from the UK Biobank and FinnGen Study, and presented genome-wide association results for sexual behavior and twelve CVDs (AP, AFF, CAS, AD, DVT-LE, HF, HTN, ICH, IS, MI, PE, and SAH). First, the UVMR design was applied to assess the total effect of AFS (both sexes) and NSP (both sexes) on CVDs. Second, we analyzed gender-stratified AFS SNPs to re-analyze the effects on CVDs to elucidate gender differences in AFS-CVD causality. Third, we conducted an MVMR design to infer the direct effects of AFS on CVD after controlling for the effect of potential confounders and mediators including TV, VPA 10 + min, and insomnia. Finally, we conducted a two-step MR to calculate the proportion of the AFS-CVD effect due to potential mediators.

## Exposures data source

Summary statistics for AFS (female, *n* = 214,547; male, *n* = 182,791) and NSP (both sexes, *n* = 370,711) were both derived from a well-powered GWAS meta-analysis in UK Biobank (18, 19) (Table 1). The UK Biobank is one of the most accessible, largest, and most intensive prospective cohort studies, collecting deep genetic and phenotypic data on approximately 500,000 individuals aged 40 to 69 years across the UK. The UK Biobank creates opportunities to explore the links between human genetic variations and diseases, as well as their association with a wide range of environmental and lifestyle factors (20). Complete predictor data were obtained from GWAS, of which only 185 AFS SNPs, 39 female AFS SNPs, 46 male AFS SNPs, and 115 NSP SNPs could be extracted from the CVD data.

**Table 1:** Overview of exposure and 12 CVD outcomes from GWAS.

Phenotype	Ancestry	Consortium	(Case/control) sample size	PMID/GWASID
Exposures				
AFS (Both sexes)	European	UK biobank	397,338	34211149
AFS (female)	European	UK biobank	214,547	34211149
AFS (male)	European	UK biobank	182,791	34211149
NSP	European	UK biobank	370,711	30643258
VPA 10 + min	European	UK biobank	440,512	ukb-b-151
Insomnia	European	UK biobank	336,965	ukb-a-13
TV	European	UK biobank	437,887	ukb-b-5192
Outcomes				
AP	European	Finn Gen	(27,046/260,124) 287,170	finn-b-I9_ANGINA
AFF	European	Finn Gen	(34,748/156,457) 191,205	finn-b-I9_AF
AD	European	Finn Gen	(680/288,638) 289,318	finn-b-I9_AORTDIS
DVT -LE	European	Finn Gen	(7,008/267,090) 274,098	finn-b-I9_PHLETHROMBDVTLOW
HF	European	Finn Gen	(19,676/272,371) 292,047	finn-b-I9_HEARTFAIL_EXNONE
HTN	European	Finn Gen	(85,438/223,663) 309,101	finn-b-I9_HYPTENS
ICH	European	Finn Gen	(2,643/281,373) 299,914	finn-b-I9_ICH
IS	European	Finn Gen	(16,857/283,057) 299,914	finn-b-I9_STR_EXH
MI	European	Finn Gen	(20,404/260,124) 280,528	finn-b-I9_MI_STRICT_EXNONE
PE	European	Finn Gen	(6,753/301,704) 308,457	finn-b-I9_PULMEMB
SAH	European	Finn Gen	(1,961/281,638) 283,599	finn-b-I9_SAH
CAS	European	Finn Gen	(23,363/187,840) 211,203	finn-b-I9_CORATHER

Overview of exposure and CVD outcomes from GWAS. CVD, cardiovascular disease; GWAS, genome-wide association study; AFS, age at first sex; AP, angina pectoris; AFF, atrial fibrillation and flutter; VPA 10 + min, number of days of vigorous physical activity 10 + minutes; CAS, coronary atherosclerosis; AD, aortic dissection; DVT-LE, deep vein thrombosis of the lower extremity; HF, heart failure; HTN, hypertension; ICH, intracerebral hemorrhage; IS, ischaemic stroke; MI, myocardial infarction; PE, pulmonary embolism; SAH, subarachnoid hemorrhage.

## Outcome data sources

Summary statistics for CVD statistics were all obtained from the FinnGen study (https://www.finngen.fi/en). The FinnGen research project is a public-private partnership that plans to collect genotype data and digital health record data from 50,000 individuals to study genetic variants associated with disease trajectories in isolated populations. The sample size of each CVD was as follows: AP (27,046 cases and 260,124 controls), AFF (34,748 cases and 156,457 controls), AD (680 cases and 288,638 controls), DVT-LE (7,008 cases and 267,090 controls), HF (19,676 cases and 272,371 controls), HTN (85,438 cases and 223,663 controls), ICH (2,643 cases and 281,373 controls), IS (16,857 cases and 283,057 controls), MI (20,404 cases and 260,124 controls), PE (6,753 cases and 301,704 controls), SAH (1,961 cases and 281,638 controls), and CAS (23,363 cases and 187,840 controls). Thus, there is no overlap between exposures and outcomes. Also, the MR design was restricted to individuals of predominantly European ancestry to minimize the bias potential for group heterogeneity. In brief, we summarized the details of the GWAS dataset in Table 1. All studies included in cited genome-wide association studies had ethical approval from their respective institutional review boards and informed consent in writing was obtained from each study participant. This study was based on publicly available datasets, and no additional ethical consent was required.

## Instrumental variables

In UVMR and MVMR, we searched for SNPs that showed a strong genome-wide statistical significance (*P *< 5.0E-08). Besides, all SNPs were independent after being removed with linkage disequilibrium (r^2^ > 0.001 or clump window <10 Mb). The *F*-statistic was used to estimate the power sizes of the IVs and was considered to have a strong effect with *F*-statistics > 10 (21).

## Statistical and sensitivity analyses

In UVMR, we used inverse variance weighted MR (IVW MR) as our main analytical method to assess the causal effects of sexual behaviors on CVDs. This IVW method provides the most accurate estimate by obtaining an estimate of the effect of each SNP on exposure and outcome risk; however, the performance of IVW is particularly sensitive to horizontal pleiotropy (22). In addition, we supplemented the MR Egger (23), the weighted median (24), weighted mode (25) and simple mode, because the cross-method results consistency ensures the robustness of the results. Directional horizontal pleiotropy was tested by MR-Egger intercept (*P* < 0.05 were considered statistically significant) and MR-PRESSO global test. The outliers (*P *< 0.05) were excluded in the MR-PRESSO global test for the significant horizontal pleiotropy, and remaining SNPs were recalculated. The degree of heterogeneity was assessed using Cochran's Q by MR-Egger estimates (*P* < 0.05 were considered statistically significant), and the “leave-one-out” sensitivity analysis was made to determine whether heterogeneity was caused by specific SNPs. In addition, the pooled results were displayed in forest plots, scatter plots, leave-on plots, and funnel plots.

MVMR, an emerging method of incorporating genetic variation for each potential confounder or mediator into the same model, can indicate potential genetic overlap between AFS and other risk factors that may lead to associated polymorphisms (26, 27).We used the MVMR IVM method to account for the effects of VPA 10 + min, TV, and insomnia of AFS on the CVD outcomes. Also, heterogeneity was quantified using a modified Cochran *Q*-statistic, and conditional IV strength was computed using the modified *F*-statistic.

In the 2-step MR analysis, we first used the UVMR to assess the separate effects of AFS on each mediator (*β*1). In addition, MVMR was used to estimate the effect of each mediator on CVDs (*β*2), while also adjusting for the genetic effect of AFS (*β*3). The coefficients method was then performed to estimate the indirect mediation effect of AFS on the CVD outcomes, i.e., the causal effect of AFS on the outcome through the mediator (*β*1 × *β*2). The total effect was the sum of the direct and indirect effects (*β*3 + *β*1 × *β*2), and the proportion of the AFS-CVD effect due to each mediator was separately computed by dividing the indirect effect by the total effect, i.e., *β*1 × *β*2 / (*β*3 + *β*1 × *β*2) (28, 29).

In general, associations between genetically predicted sexual behavior and risk of CVDs were shown as odds ratios (ORs) with 95% confidence intervals (CIs). Because this work involved a large amount of data related to exposure to the outcome, we used a uniform *P* < 0.05 to indicate statistical significance. All analyses were performed using R (version R version 4.2.1) with the “TwoSampleMR” package.

## Results

### Total effect of sexual behavior on CVDs

**Figure 1:**
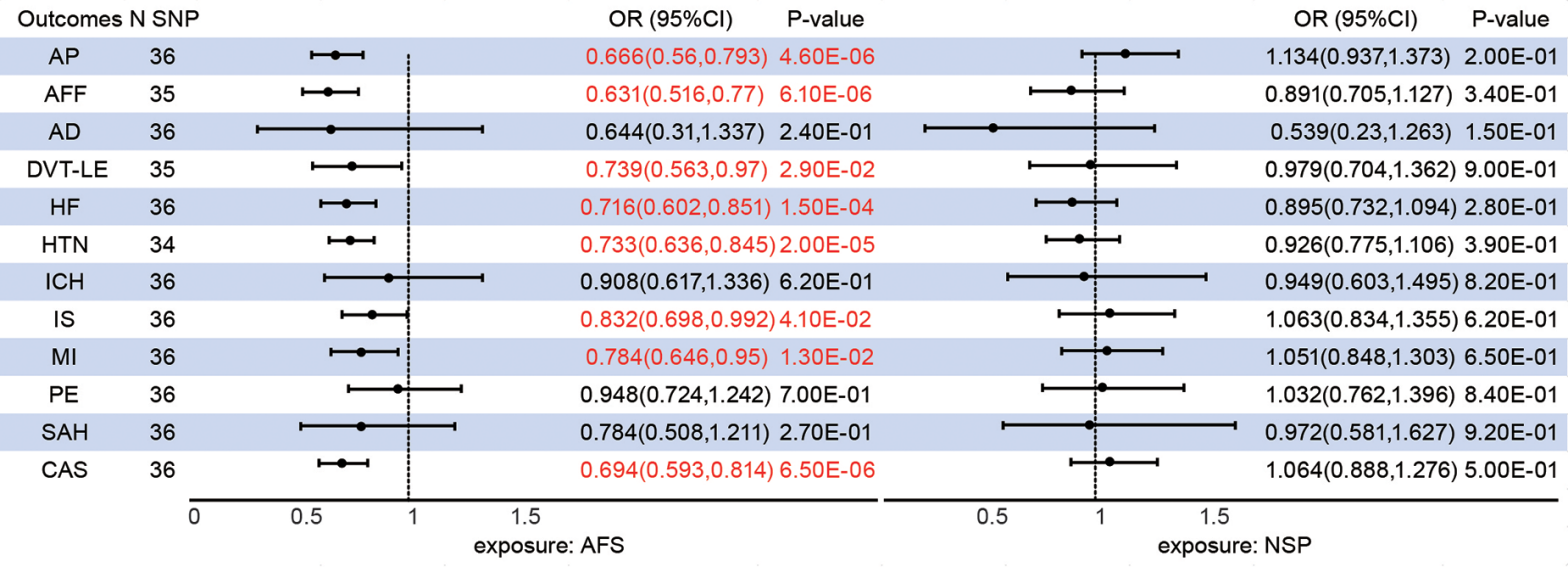
Associations between genetically predicted sexual behavior and cardiovascular diseases. AFS, age at first sex; NSP, the number of sexual partners; AP, angina pectoris; AFF, atrial fibrillation and flutter; CAS, coronary atherosclerosis; AD, aortic dissection; DVT-LE, deep vein thrombosis of the lower extremity; HF, heart failure; HTN, hypertension; ICH intracerebral hemorrhage; IS, ischaemic stroke; MI, myocardial infarction; PE, pulmonary embolism; SAH, subarachnoid hemorrhage; CI, confidence interval; SNP, single nucleotide polymorphism.

The median *F*-statistics were 17.37 for predictors of AFS and 15.04 for predictors of AFS suggesting a strong instrument (Supplementary Table S1). The primary UVMR IVW method after outlying SNP in MR-PRESSO analysis provided some evidence for negative associations of genetically predicted AFS with a wide range of CVDs, including AP [OR = 0.667, 95%CI (0.561,0.794), *P* = 4.65E-06], AFF [OR = 0.638, 95%CI (0.523,0.779), *P* = 9.55E-06], CAS [OR = 0.694, 95%CI (0.593,0.814), *P* = 6.48E-06], DVT-LE (OR = 0.739(0.563,0.97), 95%CI (0.563,0.97), *P* = 2.93E-02), HF [OR = 0.712, 95%CI (0.599,0.846), *P* = 1.12E-04], HTN [OR = 0.736, 95%CI (0.641,0.846), *P* = 1.49E-05], IS [OR = 0.832, 95%CI (0.698,0.992), *P* = 4.08E-02], and MI [OR = 0.784, 95%CI (0.646,0.95), *P* = 1.32E-02] (Figure 1). There is no evidence of a causal association of AFS on AD, ICH, PE, and SAH. Notably, no evidence of a causal relationship between NSP and CVDs.

### Total effect of gender-specific AFS on CVDs

**Figure 2:**
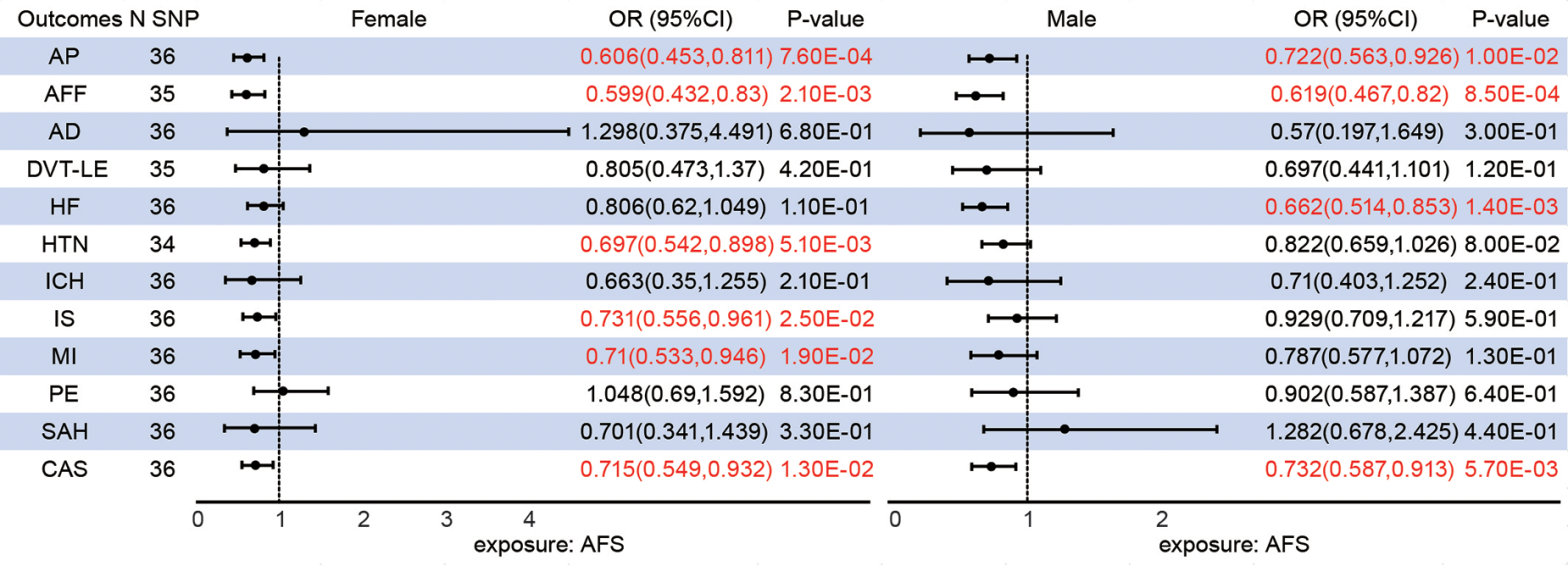
Different sex associations between genetically predicted age at first sex and cardiovascular diseases.

The median *F*-statistics were 16.53 for female AFS and 15.40 for male AFS (Supplementary Table S1). Genetically predicted female AFS has a more pronounced causal effect than male AFS on AP (female OR = 0.606; male OR = 0.722), AFF (female OR = 0.592; male OR = 0.619), and CAS (female OR = 0.715; male OR = 0.732). Furthermore, younger AFS in females can increase the risk of HTN [OR = 0.68, 95%CI (0.527,0.877), *P *= 2.99E-03], IS (OR = 0.731(0.556,0.961), 95%CI (0.556,0.961), *P* = 2.45E-02), and MI [0.71, 95%CI (0.533,0.946), *P *= 1.94E-02] in addition to HF (*P* = 1.09E-01). Conversely, male AFS was associated with HF [OR = 0.662, 95%CI (0.514,0.853), *P* = 1.42E-03] rather than HTN (*P* = 1.26E-01), IS (*P* = 5.93E-01) and MI (*P* = 1.29E-01) (Figure 2).

### Direct effects of AFS on CVDs

**Figure 3:**
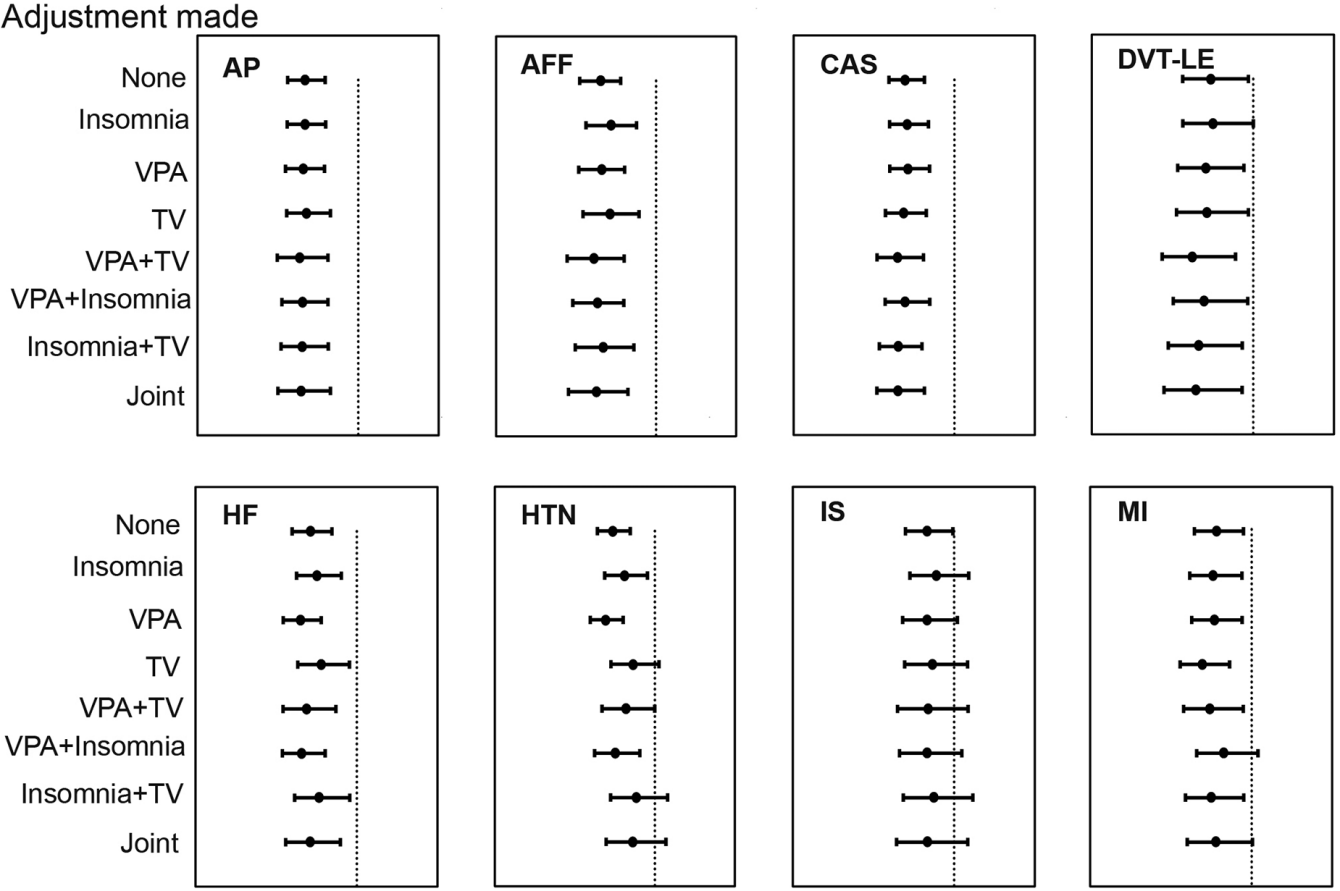
The relationship between age at first sex and cardiovascular diseases after adjusting for number of days of vigorous physical activity 10 + min and insomnia in multivariable Mendelian randomization.

Following consideration of VPA 10 + min, TV, and insomnia, we conducted MVMR IVM to assess the direct causal effects of AFS on CVDs. In addition, multiple MVMR combination models were used to account for the potential overlap of effects among the various risk factors. The protective effect of later AFS on AP, AFF, CAS, DVT-LE, and HF remained while disappearing for HTN, MI, and IS. Of these, combining any one of the other risk factors with TV was observed to diminish the effect of AFS on HTN. The causal relationship between AFS and IS disappeared in any combined MVMR models. The effect of AFS on MI disappeared in AFS-VPA 10 + min-insomnia-MI and joint model (Figure 3, Supplementary Table S1).

### Mediation analysis

First, primary UVMR IVW methods showed genetically predicted AFS was associated with insomnia [OR = 0.885, 95%CI (0.857,0.913), *P* = 6.76E-14], VPA 10 + min [OR = 0.833, 95%CI (0.775,0.895), *P *= 5.68E-07] and TV [OR = 0.812, 95%CI (0.785,0.841), *P* = 2.37E-32]. Second, in the previous MVMR analysis, we estimated the effect of each mediator on eight CVDs, while also adjusting for the genetic effect of AFS. In summary, the effect of AFS on HF and HTN was partially mediated by insomnia (proportion mediated, 19.89% for HF, 25.00% for HTN) and VPA 10 + min (proportion mediated, 19.92% for HF, 25.15% for HTN). Furthermore, the proportion of mediators for television in the causal effects of AFS-AP and AFS-HTN was 16.90% and 25.46%, respectively (Table 2).

**Table 2:** Results of mediation analysis in two-step MR.

Potential mediators	Outcomes	AFS to mediators	*P*-value (AFS to mediators)	Mediators to CVDs	*P*-value (mediators to CVDs)	Direct AFS to CVDs (mediator adjusted)	*P*-value (direct AFS to outcomes)	% Mediation (indirect)
Insomnia	AP	−0.1227	6.76E-14	−0.4055	3.74E-01	−0.4055	8.63E-06	
	AFF	−0.1227	6.76E-14	0.2306	5.01E-01	−0.3533	1.87E-03	
	CAS	−0.1227	6.76E-14	0.1981	4.48E-01	−0.3461	6.27E-05	
	DVT-LE	−0.1227	6.76E-14	0.3946	3.73E-01	−0.2860	5.11E-02	
	HF	−0.1227	6.76E-14	0.5741	4.13E-02	−0.2837	2.32E-03	19.89
	HTN	−0.1227	6.76E-14	0.5749	2.36E-02	−0.2117	1.18E-02	25.00
	IS	−0.1227	6.76E-14	0.2664	3.95E-01	−0.1170	2.59E-01	
	MI	−0.1227	6.76E-14	0.4450	1.70E-01	−0.2717	1.15E-02	
VPA 10 + min	AP	−0.1831	5.68E-07	0.0406	8.00E-01	−0.4189	7.00E-06	
	AFF	−0.1831	5.68E-07	−0.2690	1.65E-01	−0.4385	9.75E-05	
	CAS	−0.1831	5.68E-07	0.0867	5.69E-01	−0.3411	1.16E-04	
	DVT-LE	−0.1831	5.68E-07	−0.0289	9.08E-01	−0.3458	1.77E-02	
	HF	−0.1831	5.68E-07	−0.3897	1.38E-02	−0.4296	2.91E-06	19.92
	HTN	−0.1831	5.68E-07	−0.4048	1.91E-03	−0.3689	1.12E-06	25.15
	IS	−0.1831	5.68E-07	−0.2544	1.51E-01	−0.1823	7.64E-02	
	MI	−0.1831	5.68E-07	0.2645	1.35E-01	−0.2612	1.11E-02	
TV	AP	−0.2080	2.37E-32	0.3807	2.76E-02	−0.3896	1.27E-04	16.90
	AFF	−0.2080	2.37E-32	0.1158	5.92E-01	−0.3619	4.36E-03	
	CAS	−0.2080	2.37E-32	0.2403	1.32E-01	−0.3764	6.00E-05	
	DVT-LE	−0.2080	2.37E-32	−0.0994	7.09E-01	−0.3375	3.10E-02	
	HF	−0.2080	2.37E-32	0.4126	1.97E-02	−0.2513	1.57E-02	25.46
	HTN	−0.2080	2.37E-32	0.5966	7.16E-05	−0.1478	9.45E-02	
	IS	−0.2080	2.37E-32	0.0946	6.25E-01	−0.1438	2.07E-01	
	MI	−0.2080	2.37E-32	0.2562	1.78E-01	−0.3648	1.10E-03	

Results of mediation analysis in two-step MR. AFS, age at first sex; AP, angina pectoris; AFF, atrial fibrillation and flutter; CAS, coronary atherosclerosis; DVT-LE, deep vein thrombosis of the lower extremity; HF, heart failure; HTN, hypertension; IS, ischaemic stroke; MI, myocardial infarction; VPA 10 + min, number of days of vigorous physical activity 10 + minutes.

### Sensitivity analysis

In UVMR analysis, the results of MR sensitivity methods were consistent, even if the range of partial confidence intervals was extended (Supplementary Table S2). No directional horizontal pleiotropy polymorphism was detected with MR-Egger intercept (Supplementary Table S3). Nevertheless, Cochran's *Q* by MR-Egger estimates showed mild to medium heterogeneity in certain outcomes **(**Supplementary Table S4**)**. In the MR-PRESSO global test, we identified outlier SNPs and detailed the remaining SNPs and outliers in Supplementary Tables S5-S8. Detailed forest plots, scatter plots, leave-on plots, and funnel plots could be visualized in supplementary Figure S1–S16. For MVMR, modified Cochran's *Q*-statistic likewise demonstrated partial heterogeneity (Supplementary Table S1).

## Discussion

In the present study, we systematically analyzed the causal effects between two sexual behaviors and twelve CVDs using two-sample UVMR, MVMR, and two-step MR, respectively. First, the results of our UVMR analysis showed that genetically predicted AFS were significantly causally associated with eight CVDs (AP, AFF, CAS, DVT-LE, HF, HTN, IS, and MI), which were consistent with the previous findings. Also, there was no evidence to support a causal effect between NSP and any CVD. Next, given the possible gender differences in AFS on CVDs, we used gender-stratified SNPs to investigate. The results showed a stronger causal association of female AFS on CVDs than male. Then, MVMR analysis revealed that AFS remained causally related to CVDs after accounting for VPA 10 + min, insomnia, and TV. Finally, mediation MR analyses further demonstrated that AFS mediated the effect on individual CVDs through three risk factors separately.

The results of our study were consistent with previous MR studies, which reported a consistent association between early AFS and the risk of a wide range of CVDs. In contrast to existing observational studies (12), we also had the new finding that later AFS had a protective effect on the risk of AP, AFF, CAS, DVT-LE, HF, IS, and MI, which was an important supplement to the causal effect of AFS on CVDs. MVMR analyses showed that the effects of AFS on HTN and IS were attenuated after controlling for VPA 10 + min, insomnia, and TV, but remained on other outcomes. These results again emphasized the potential pleiotropic influence in the genetic instruments of AFS. Notably, we also reported the effect of VPA 10 + min, insomnia, and TV on CVD outcomes. These results pointed to a mediation and co-activation role of VPA 10 + min, insomnia, and TV in the causal relationship of AFS on HF, AP, and HTN.

The Developmental Origins of Health and Disease (DOHaD) hypothesis proposes that adverse environmental exposures during early life lead to developing chronic disease in adulthood (30). For example, trauma during key developmental periods, especially before the age of 16, is considered an accelerant for CVD development (16). Thus, early sexual intercourse may have a profound negative long-term effect on health. In fact, recent studies focus on sexual healthcare and sexual function in patients with CVDs, but they neglected the role of sexuality in the development of CVDs. The underlying mechanisms of the association between AFS and CVD have not been fully described. More research is necessary for the future to explore the complex relationship between sexual behavior and CVD outcomes.

For the gender-stratified AFS-CVDs study, female AFS appears to have a broader impact on CVD trajectories and outcomes than male AFS. The self-reported earlier age at first sex of women, rather than men, has a tendency to be forced (31). In addition, women are subject to more negative emotions for their first sexual encounter (32).A meta-analytic review reports that childhood abuse has a powerful association with an increased risk of disease of CVDs in adulthood, with a greater impact on women (33). The stress response system may be chronically activated as a result of adverse childhood experiences, contributing to autonomic, neuroendocrine and inflammatory dysfunctions and subsequent development of CVD (34). Nevertheless, the mechanisms involved are worthy of further clarification.

In this perspective, addressing sexual abuse in childhood and adolescence, delaying the age of first sexual intercourse and psychological support for those who start first sexual intercourse earlier are important for the prevention and management of reproductive health and cardiovascular disease. There was evidence that comprehensive sexuality education in schools is associated with a delay in the age of first sexual intercourse (35). At the same time, out-of-school comprehensive sexuality education was critical in order for certain non-educated teens to know their bodies, health, and sexual behavior (36). Additionally, improving sleep quality as well as substituting physical activity for sedentary behavior and TV watching can be effective in promoting cardiovascular health.

### Strengths and limitations

The overriding advantage was the two-sample MR study design, which minimized the reverse causality and remaining confounders of observational studies, while being less time-consuming, less costly, and more accessible than randomized controlled trials. Two-sample MR, i.e., exposure and outcome data from different samples, makes the results more reliable, and the “winner's curse” (37) can skew the true causal effects to favor the observed outcomes in one-sample MR. Additionally, we obtained the AFS and NSP IVs from the largest, most up-to-date GWAS data to ensure adequate power for the IVs in the MR analysis. We hierarchically used MVMR, UVMR, and two-step MR to comprehensively explore a broader range of CVDs and risk factors. Sex-specific SNPs were utilized on the outcome to demonstrate the role of sex in the development of AFS-CVDs. Finally, all studies were confined to European participants to minimize population stratification bias.

Inevitably, a few limitations are in the present study. Several conditional F-statistics in MVMR models were less than 10, suggesting a risk of the weakness of the instrument, which may contribute to inaccurate estimates of the direct effect and mediation effect of AFS on CVDs. In the sex-specific MR analysis, we used sex-stratified exposures, but we were unavailable for sex-specific CVDs data, which could lead to collider bias (38). What's more, the coefficients method was used to estimate the proportion of the AFS-CVD effect through each risk factor individually. Nevertheless, this approach is not currently available for simultaneous consideration of multiple mediators in the MR model. Finally, there may be racial heterogeneity in the negative effect of AFS on CVDs such as hypertension (12), and the study only used European populations, more racial studies are needed to complement our results.

## Conclusion

To conclude, this study using distinct MR analytical methods showed evidence that earlier AFS rather than NSP was associated with an increased risk of CVDs. Early female sexual behavior more pervasively affects the trajectory and outcomes of CVDs than early male sexual behavior. Less VPA 10 + min, insomnia, and TV playing mediator or common roles in AFS-CVDs. Interfering with these risk factors may serve as a new strategy for preventing and managing CVDs.

## Data Availability

The original contributions presented in the study are included in the article/**Supplementary Material**, further inquiries can be directed to the corresponding authors.

## References

[1] Diseases GBD, Injuries C. Global burden of 369 diseases and injuries in 204 countries and territories, 1990–2019: a systematic analysis for the global burden of disease study 2019. Lancet. (2020) 396(10258):1204–22. 10.1016/S0140-6736(20)30925-9 33069326 PMC7567026

[2] Joseph P Leong D McKee M Anand SS Schwalm JD Teo K Reducing the global burden of cardiovascular disease, part 1: the epidemiology and risk factors. Circ Res. (2017) 121(6):677–94. 10.1161/CIRCRESAHA.117.308903 28860318

[3] Roth GA Mensah GA Johnson CO Addolorato G Ammirati E Baddour LM Global burden of cardiovascular diseases and risk factors, 1990-2019: update from the GBD 2019 study. J Am Coll Cardiol. (2020) 76(25):2982–3021. 10.1016/j.jacc.2020.11.010 33309175 PMC7755038

[4] Andersson C Vasan RS. Epidemiology of cardiovascular disease in young individuals. Nat Rev Cardiol. (2018) 15(4):230–40. 10.1038/nrcardio.2017.154 29022571

[5] Dahabreh IJ Paulus JK. Association of episodic physical and sexual activity with triggering of acute cardiac events: systematic review and meta-analysis. JAMA. (2011) 305(12):1225–33. 10.1001/jama.2011.336 21427375 PMC5479331

[6] Moller J Ahlbom A Hulting J Diderichsen F de Faire U Reuterwall C Sexual activity as a trigger of myocardial infarction. A case-crossover analysis in the Stockholm heart epidemiology programme (SHEEP). Heart. (2001) 86(4):387–90. 10.1136/heart.86.4.387 11559674 PMC1729949

[7] Mota NP Cox BJ Katz LY Sareen J. Relationship between mental disorders/suicidality and three sexual behaviors: results from the national comorbidity survey replication. Arch Sex Behav. (2010) 39(3):724–34. 10.1007/s10508-008-9463-5 19219545

[8] Yuh T Micheni M Selke S Oluoch L Kiptinness C Magaret A Sexually transmitted infections among Kenyan adolescent girls and young women with limited sexual experience. Front Public Health. (2020) 8:303. 10.3389/fpubh.2020.00303 32766197 PMC7381162

[9] Antonsson A de Souza MMA Panizza BJ Whiteman DC. Sexual debut and association with oral human papillomavirus infection, persistence and oropharyngeal cancer—an analysis of two Australian cohorts. Int J Cancer. (2022) 151(5):764–9. 10.1002/ijc.33986 35225359

[10] Jian ZY Ye DH Chen YT Li H Wang KJ. Sexual activity and risk of prostate cancer: a dose-response meta-analysis. J Sex Med. (2018) 15(9):1300–9. 10.1016/j.jsxm.2018.07.004 30122473

[11] Plummer M Peto J Franceschi S Epidemiological IC. Time since first sexual intercourse and the risk of cervical cancer. Int J Cancer. (2012) 130(11):2638–44. 10.1002/ijc.26250 21702036 PMC3982220

[12] Ngueta G Ndjaboue R. Early sexual experience and hypertension in US adults: results from the national health and nutrition examination survey 2001–2016. J Hypertens. (2018) 36(12):2414–9. 10.1097/HJH.0000000000001821 29957720

[13] Davey Smith G Ebrahim S. What can Mendelian randomisation tell US about modifiable behavioural and environmental exposures? Br Med J. (2005) 330(7499):1076–9. 10.1136/bmj.330.7499.1076 15879400 PMC557238

[14] Nikpay M Mohammadzadeh S. Phenome-wide screening for traits causally associated with the risk of coronary artery disease. J Hum Genet. (2020) 65(4):371–80. 10.1038/s10038-019-0716-z 31907388

[15] Zhang XQ Yang YX Zhang C Leng XY Chen SD Ou YN Validation of external and internal exposome of the findings associated to cerebral small vessel disease: a Mendelian randomization study. J Cereb Blood Flow Metab. (2022) 42(6):1078–90. 10.1177/0271678X221074223 35018869 PMC9125490

[16] O'Neil A Scovelle AJ Milner AJ Kavanagh A. Gender/sex as a social determinant of cardiovascular risk. Circulation. (2018) 137(8):854–64. 10.1161/CIRCULATIONAHA.117.028595 29459471

[17] Lew-Starowicz M. Sexuality and sleep disorders. J Sex Med. (2022) 19(6):890–4. 10.1016/j.jsxm.2022.02.011 35304849

[18] Karlsson Linner R Biroli P Kong E Meddens SFW Wedow R Fontana MA Genome-wide association analyses of risk tolerance and risky behaviors in over 1 million individuals identify hundreds of loci and shared genetic influences. Nat Genet. (2019) 51(2):245–57. 10.1038/s41588-018-0309-3 30643258 PMC6713272

[19] Mills MC Tropf FC Brazel DM van Zuydam N Vaez A, eQTLGen Consortium, et al. Identification of 371 genetic variants for age at first sex and birth linked to externalising behaviour. Nat Hum Behav. (2021) 5(12):1717–30. 10.1038/s41562-021-01135-3 34211149 PMC7612120

[20] Bycroft C Freeman C Petkova D Band G Elliott LT Sharp K The UK biobank resource with deep phenotyping and genomic data. Nature. (2018) 562(7726):203–9. 10.1038/s41586-018-0579-z 30305743 PMC6786975

[21] Brion MJ Shakhbazov K Visscher PM. Calculating statistical power in Mendelian randomization studies. Int J Epidemiol. (2013) 42(5):1497–501. 10.1093/ije/dyt179 24159078 PMC3807619

[22] Burgess S Bowden J Fall T Ingelsson E Thompson SG. Sensitivity analyses for robust causal inference from Mendelian randomization analyses with multiple genetic variants. Epidemiology. (2017) 28(1):30–42. 10.1097/EDE.0000000000000559 27749700 PMC5133381

[23] Bowden J Smith GD Burgess S. Mendelian randomization with invalid instruments: effect estimation and bias detection through egger regression. Int J Epidemiol. (2015) 44(2):512–25. 10.1093/ije/dyv080 26050253 PMC4469799

[24] Bowden J Smith GD Haycock PC Burgess S. Consistent estimation in Mendelian randomization with some invalid instruments using a weighted median estimator. Genet Epidemiol. (2016) 40(4):304–14. 10.1002/gepi.21965 27061298 PMC4849733

[25] Hartwig FP Smith GD Bowden J. Robust inference in summary data Mendelian randomization via the zero modal pleiotropy assumption. Int J Epidemiol. (2017) 46(6):1985–98. 10.1093/ije/dyx102 29040600 PMC5837715

[26] Sanderson E Davey Smith G Windmeijer F Bowden J. An examination of multivariable Mendelian randomization in the single-sample and two-sample summary data settings. Int J Epidemiol. (2019) 48(3):713–27. 10.1093/ije/dyy262 30535378 PMC6734942

[27] Burgess S Thompson SG. Multivariable Mendelian randomization: the use of pleiotropic genetic variants to estimate causal effects. Am J Epidemiol. (2015) 181(4):251–60. 10.1093/aje/kwu283 25632051 PMC4325677

[28] Xu M Zheng J Hou T Lin H Wang T Wang S SGLT2 inhibition, choline metabolites, and cardiometabolic diseases: a mediation Mendelian randomization study. Diabetes Care. (2022) 45(11):2718–28. 10.2337/dc22-0323 36161993 PMC9862376

[29] Zhang J Chen Z Parna K van Zon SKR Snieder H Thio CHL. Mediators of the association between educational attainment and type 2 diabetes mellitus: a two-step multivariable Mendelian randomisation study. Diabetologia. (2022) 65(8):1364–74. 10.1007/s00125-022-05705-6 35482055 PMC9283137

[30] Barker DJ Osmond C. Infant mortality, childhood nutrition, and ischaemic heart disease in England and Wales. Lancet. (1986) 1(8489):1077–81. 10.1016/S0140-6736(86)91340-1 2871345

[31] Dickson N Paul C Herbison P Silva P. First sexual intercourse: age, coercion, and later regrets reported by a birth cohort. Br Med J. (1998) 316(7124):29–33. 10.1136/bmj.316.7124.29 9451263 PMC2665316

[32] Moreau N Kolto A Young H Maillochon F Godeau E. Negative feelings about the timing of first sexual intercourse: findings from the health behaviour in school-aged children study. Int J Public Health. (2019) 64(2):219–27. 10.1007/s00038-018-1170-y 30456468

[33] Wegman HL Stetler C. A meta-analytic review of the effects of childhood abuse on medical outcomes in adulthood. Psychosom Med. (2009) 71(8):805–12. 10.1097/PSY.0b013e3181bb2b46 19779142

[34] Godoy LC Frankfurter C Cooper M Lay C Maunder R Farkouh ME. Association of adverse childhood experiences with cardiovascular disease later in life: a review. JAMA Cardiol. (2021) 6(2):228–35. 10.1001/jamacardio.2020.6050 33263716

[35] Ramirez-Villalobos D Monterubio-Flores EA Gonzalez-Vazquez TT Molina-Rodriguez JF Ruelas-Gonzalez MG Alcalde-Rabanal JE. Delaying sexual onset: outcome of a comprehensive sexuality education initiative for adolescents in public schools. BMC Public Health. (2021) 21(1):1439. 10.1186/s12889-021-11388-2 34289834 PMC8296525

[36] Boborakhimov S Mosisa HB Demerew D Narenjiha M Sanjuanelo Jimenez JM Payares Lugo LE The design and delivery of out-of-school comprehensive sexuality education from the perspective of the young people it is intended to serve. Sex Reprod Health Matters. (2023) 31(2):2208769. 10.1080/26410397.2023.2208769 37341689 PMC10286664

[37] Lawlor DA. Commentary: two-sample Mendelian randomization: opportunities and challenges. Int J Epidemiol. (2016) 45(3):908–15. 10.1093/ije/dyw127 27427429 PMC5005949

[38] Holmberg MJ Andersen LW. Collider bias. JAMA. (2022) 327(13):1282–3. 10.1001/jama.2022.1820 35285854

