# Supplementary material for: Sexual behavior and cardiovascular diseases: univariable and multivariable Mendelian randomization
Source: Front Cardiovasc Med. 2023 Dec 5;10:1250404. doi: 10.3389/fcvm.2023.1250404 (PMC10728475; doi:10.3389/fcvm.2023.1250404)
Supplement: Supplementary file 1 [file Datasheet1.docx]

Supplemental Material

| **Figures** |  |
| --- | --- |
| Figure S1. Scatter plot of AFS on cardiovascular disease | Page 2 |
| Figure S2. Funnel plot of AFS on cardiovascular disease | Page 3 |
| Figure S3. Leave-one-out plot of AFS on cardiovascular disease | Page 4  Page 5 |
| Figure S4. Forest plot of AFS on cardiovascular disease | Page 6  Page 7 |
| Figure S5. Scatter plot of female AFS on cardiovascular disease | Page 8 |
| Figure S6. Funnel plot of female AFS on cardiovascular disease | Page 9 |
| Figure S7. Leave-one-out plot of female AFS on cardiovascular disease | Page 10 |
| Figure S8. Forest plot of female AFS on cardiovascular disease | Page 11 |
| Figure S9. Scatter plot of male AFS on cardiovascular disease | Page 12 |
| Figure S10. Funnel plot of male AFS on cardiovascular disease | Page 13 |
| Figure S11. Leave-one-out plot of male AFS on cardiovascular disease | Page 14 |
| Figure S12. Forest plot of male AFS on cardiovascular disease | Page 15 |
| Figure S13. Scatter plot of NSP on cardiovascular disease | Page 16 |
| Figure S14. Funnel plot of NSP on cardiovascular disease | Page 17 |
| Figure S15. Leave-one-out plot of NSP on cardiovascular disease | Page 18  Page 19 |
| Figure S16. Forest plot of NSP on cardiovascular disease | Page 20  Page 21 |
| Legends for all figures | Page 22 |

Figure S1. Scatter plot of AFS on cardiovascular disease


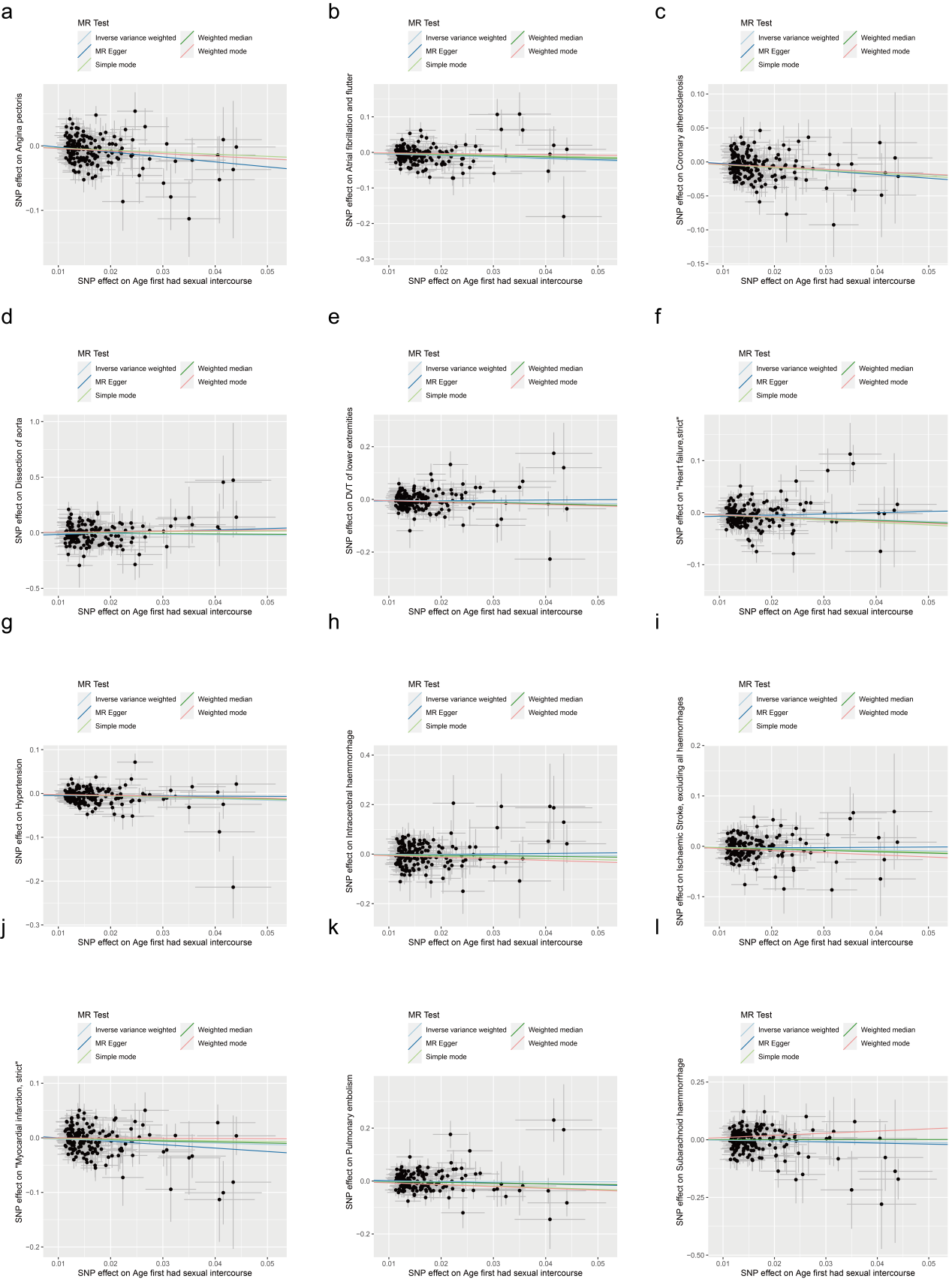


Figure S2. Funnel plot of AFS on cardiovascular disease


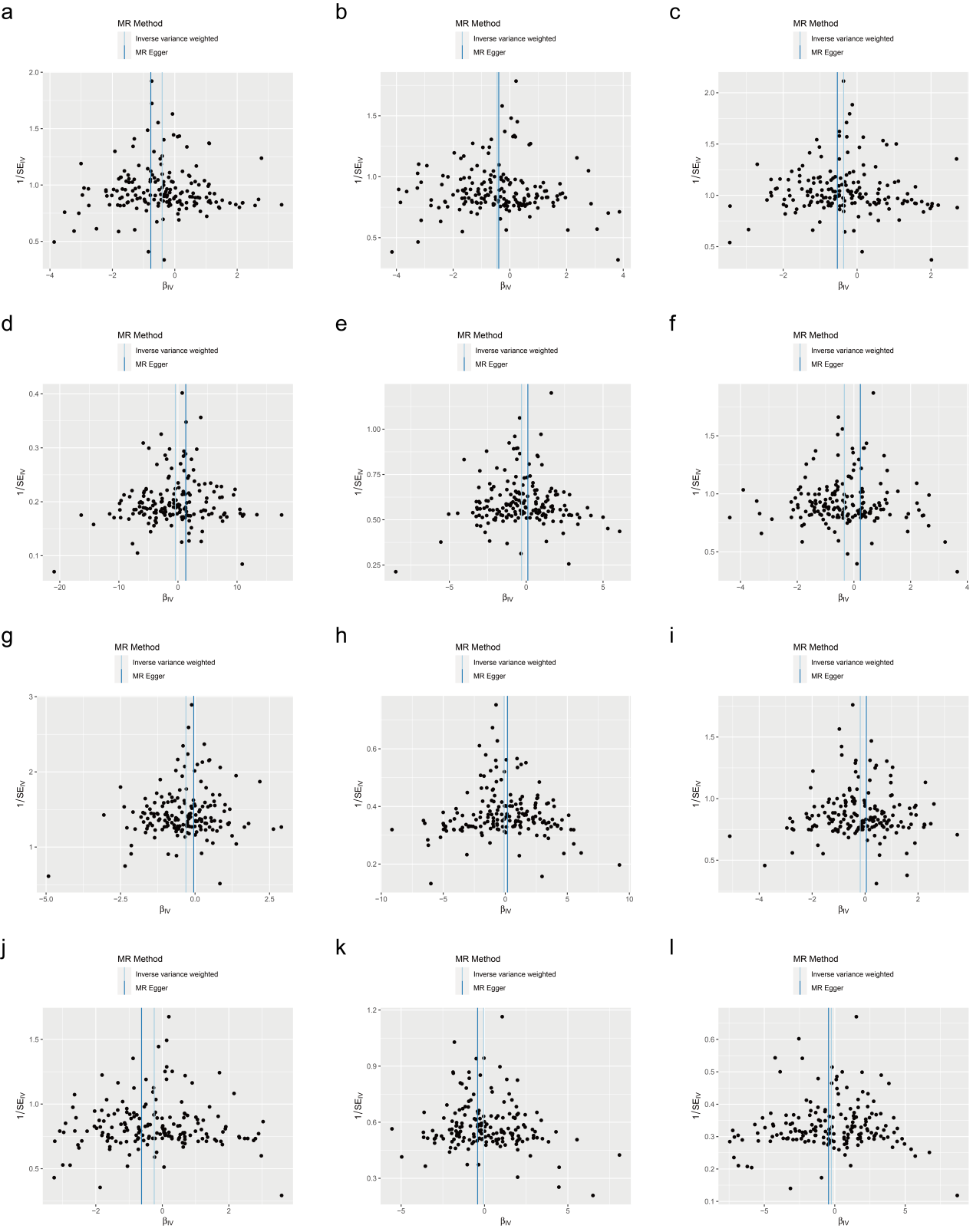


Figure S3. Leave-one-out plot of AFS on cardiovascular disease


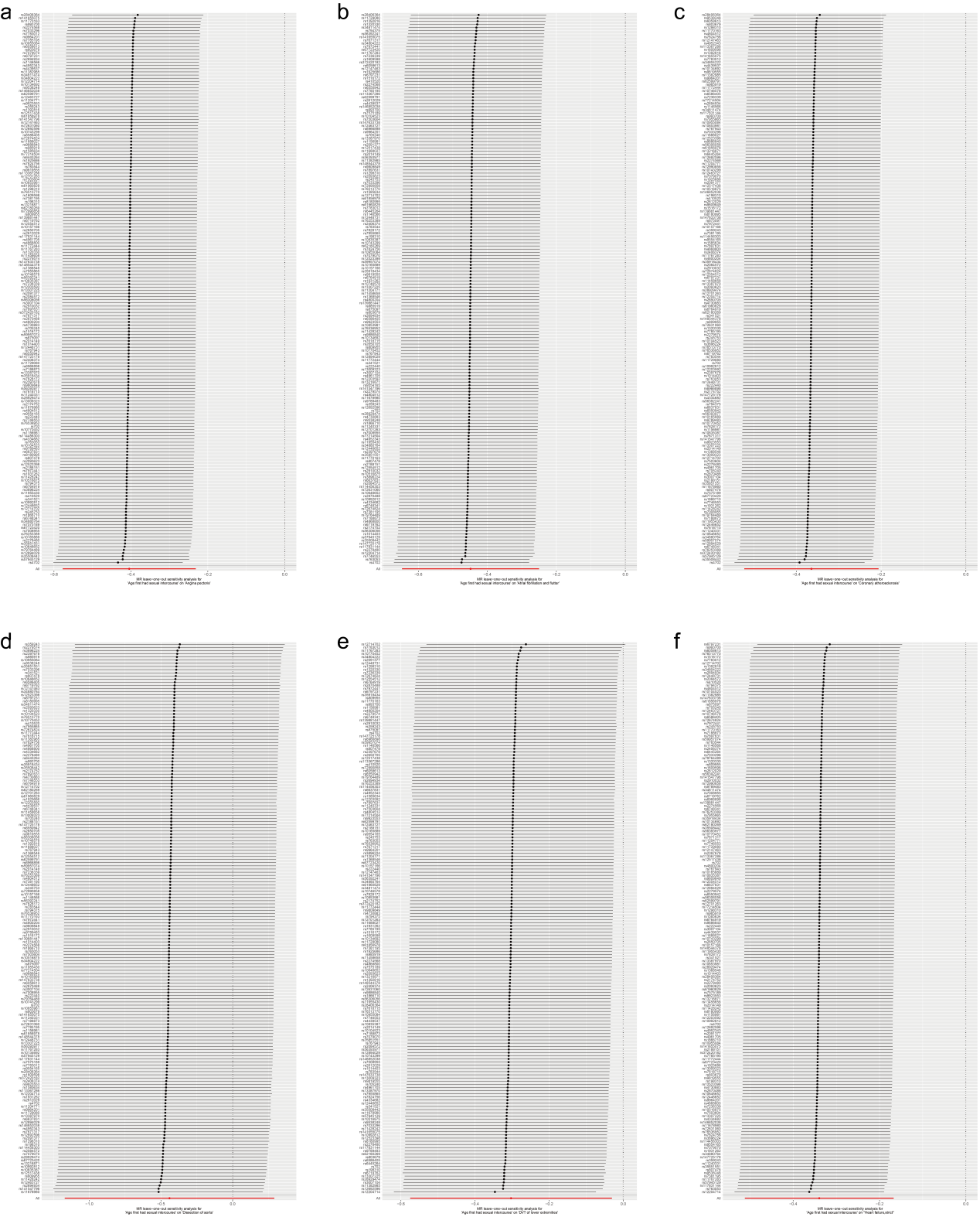


Figure S3. continued


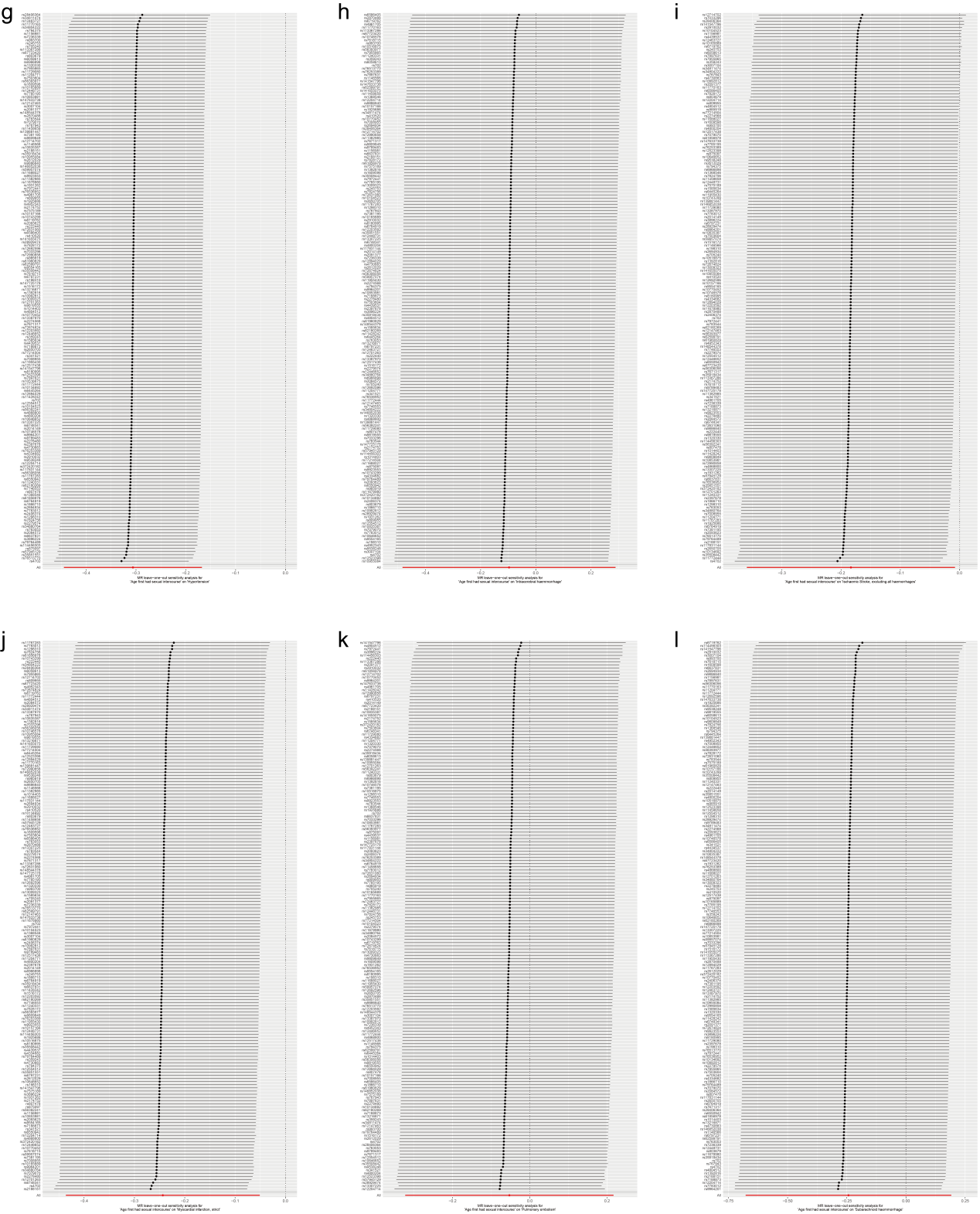


Figure S4. Forest plot of AFS on cardiovascular disease


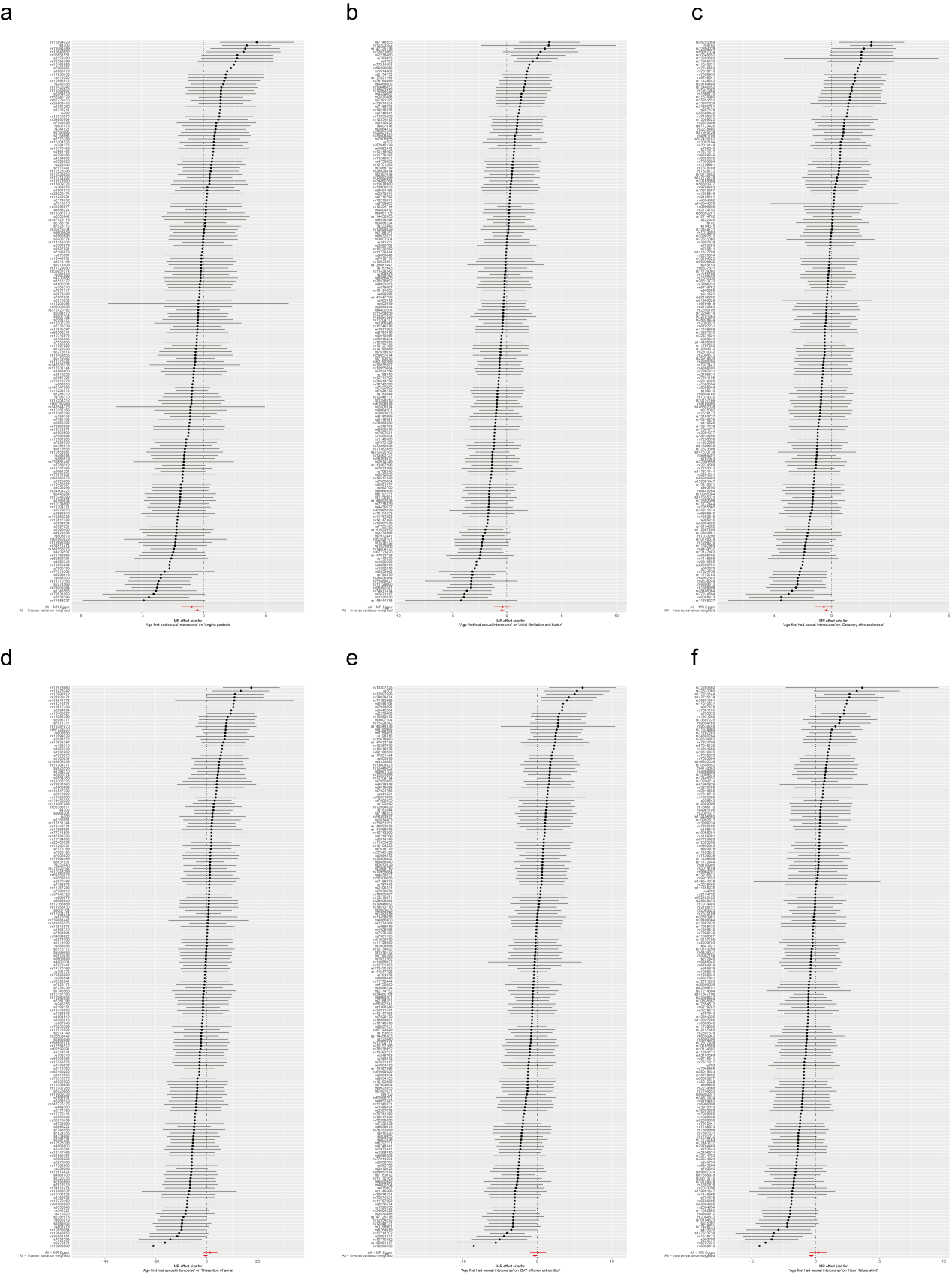


Figure S4. continued





Figure S5. Scatter plot of female AFS on cardiovascular disease


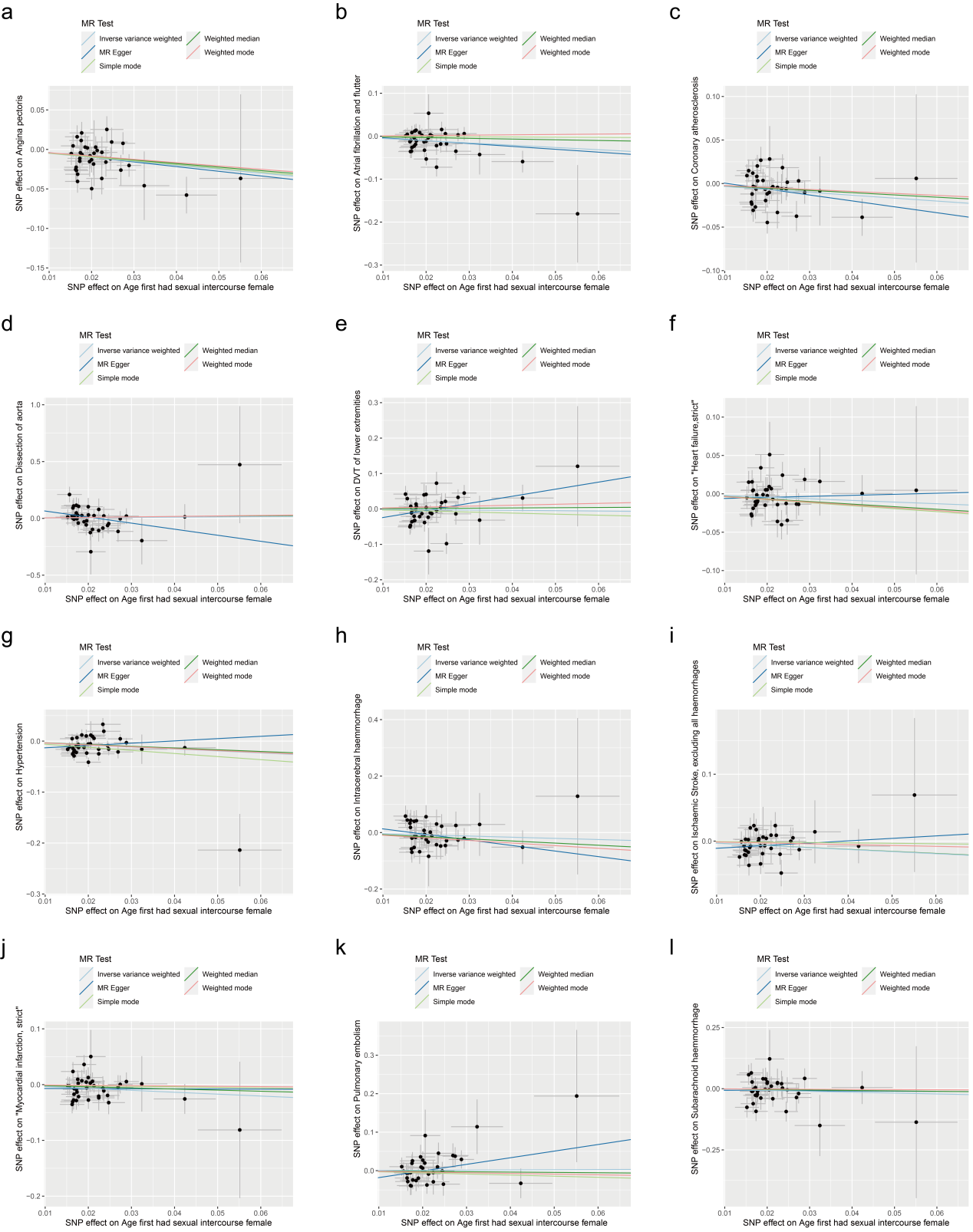


Figure S6. Funnel plot of female AFS on cardiovascular disease


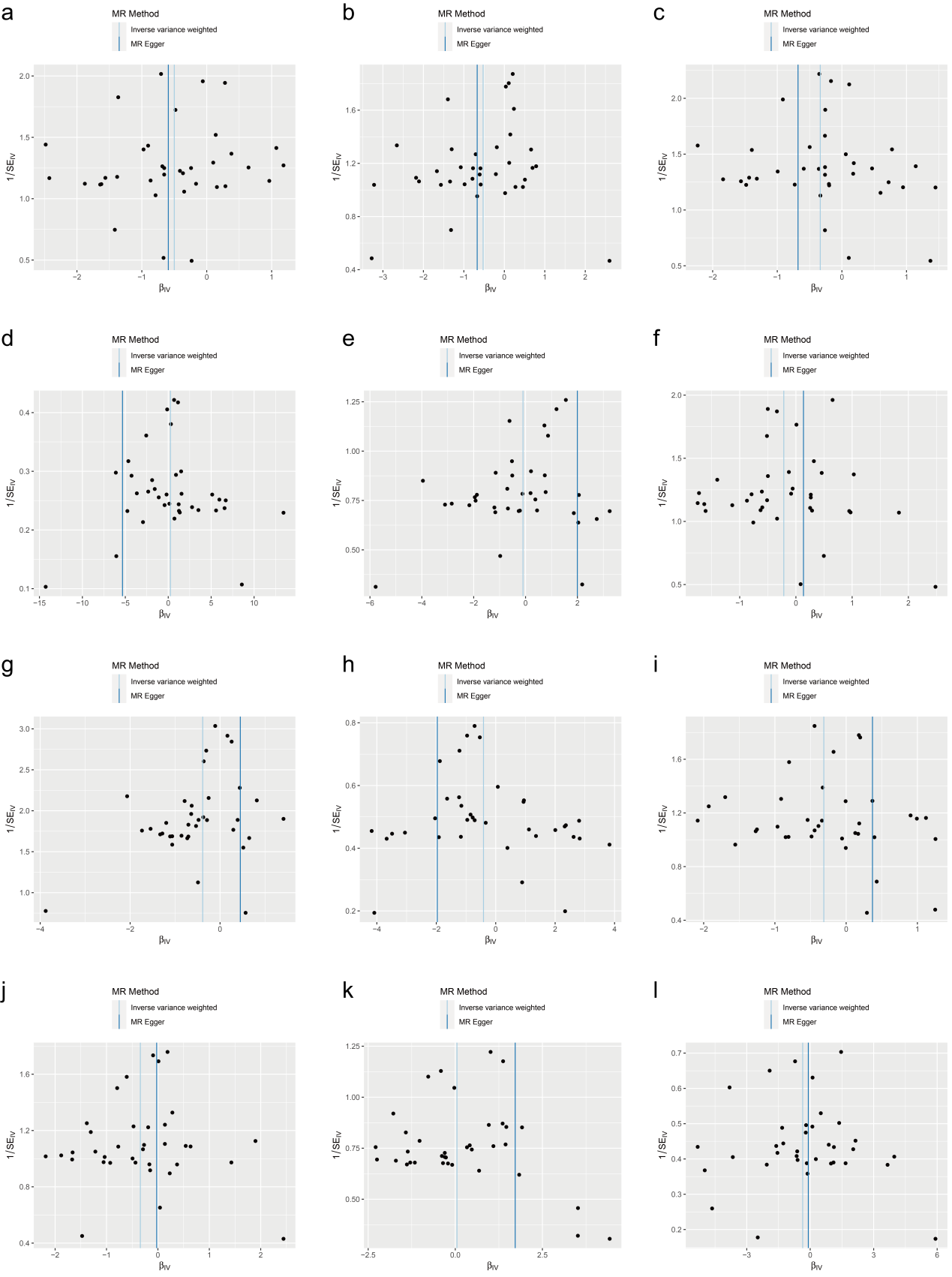


Figure S7. Leave-one-out plot of female AFS on cardiovascular disease


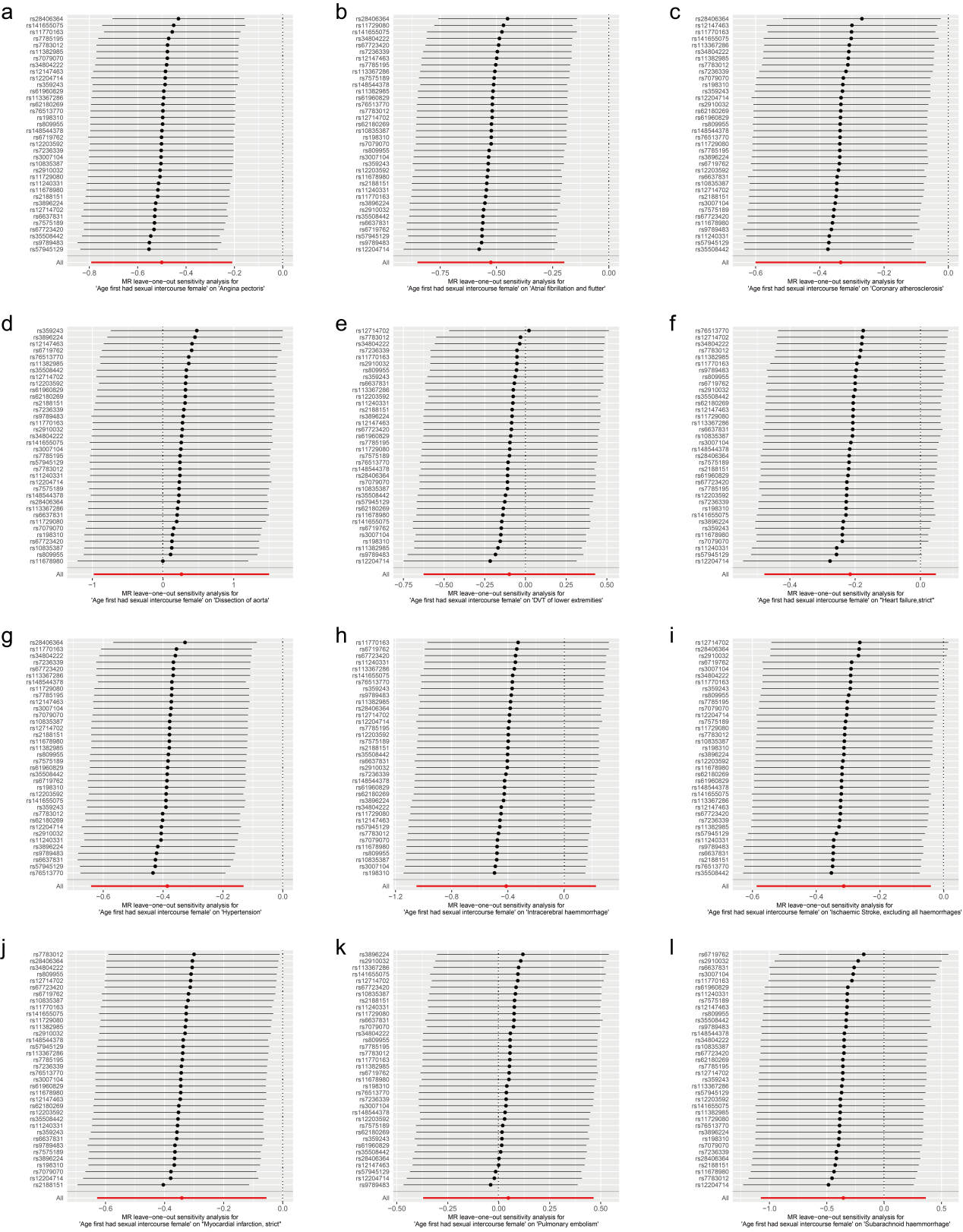


Figure S8. Forest plot of female AFS on cardiovascular disease


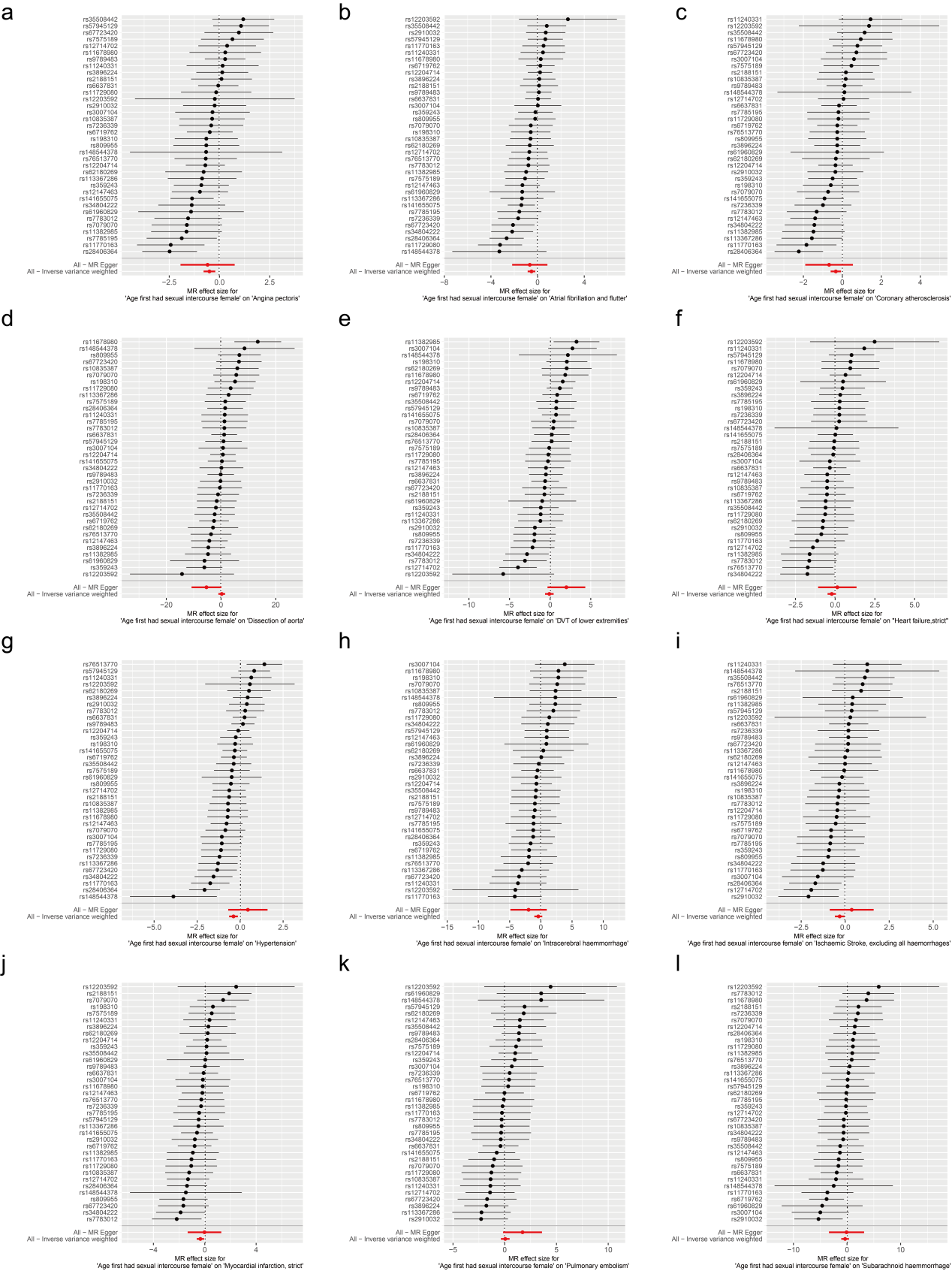


Figure S9. Scatter plot of male AFS on cardiovascular disease


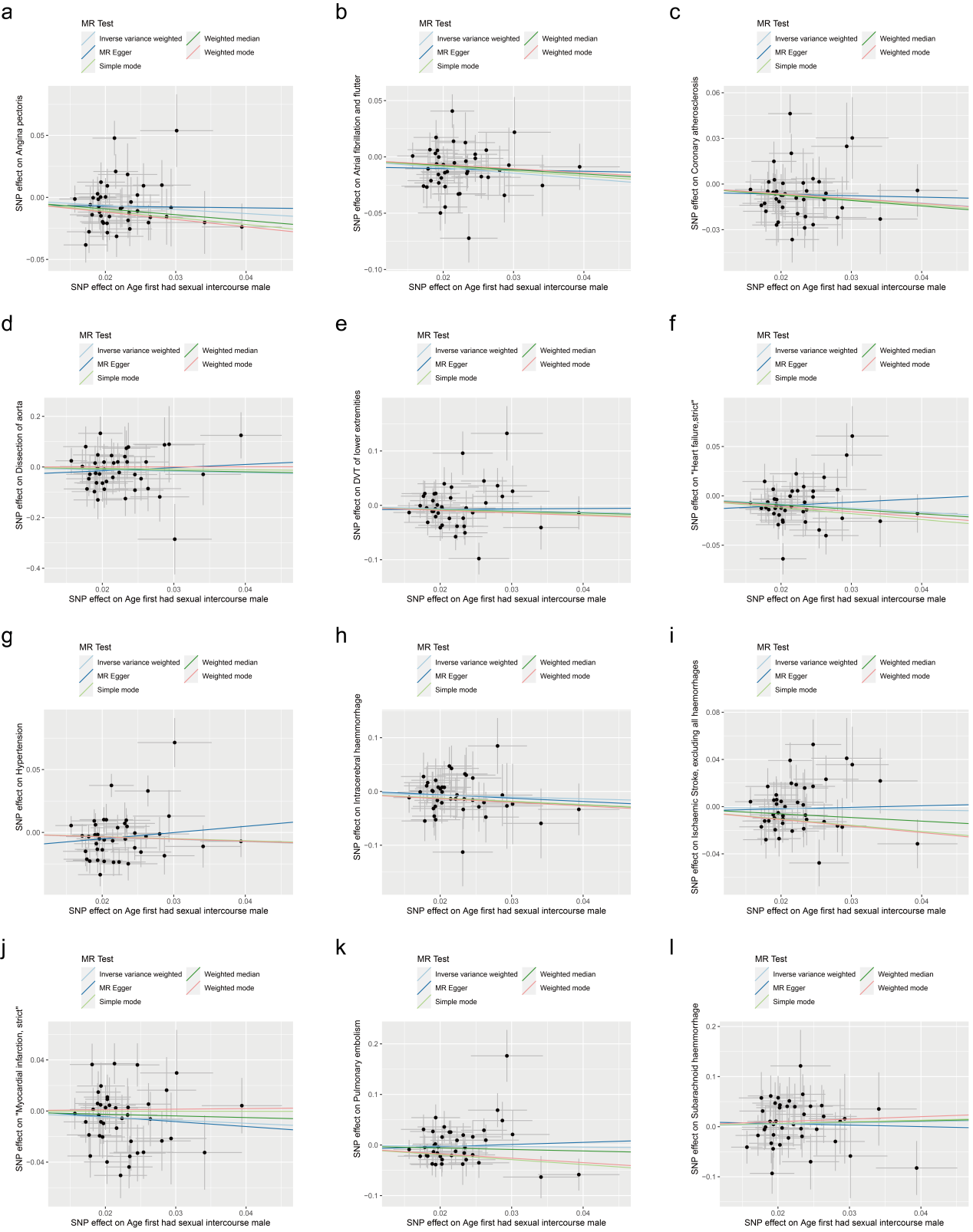


Figure S10. Funnel plot of male AFS on cardiovascular disease


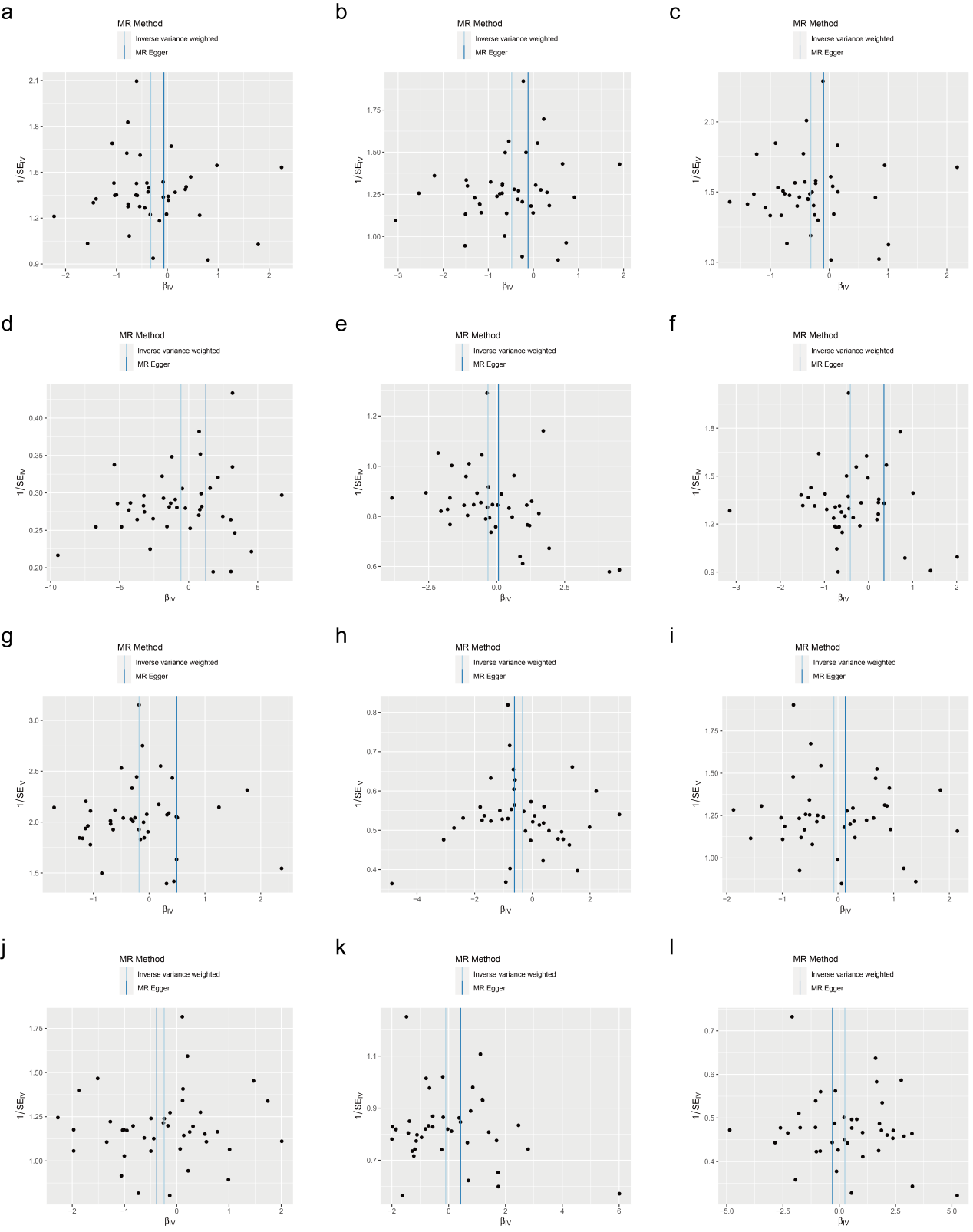


Figure S11. Leave-one-out plot of male AFS on cardiovascular disease


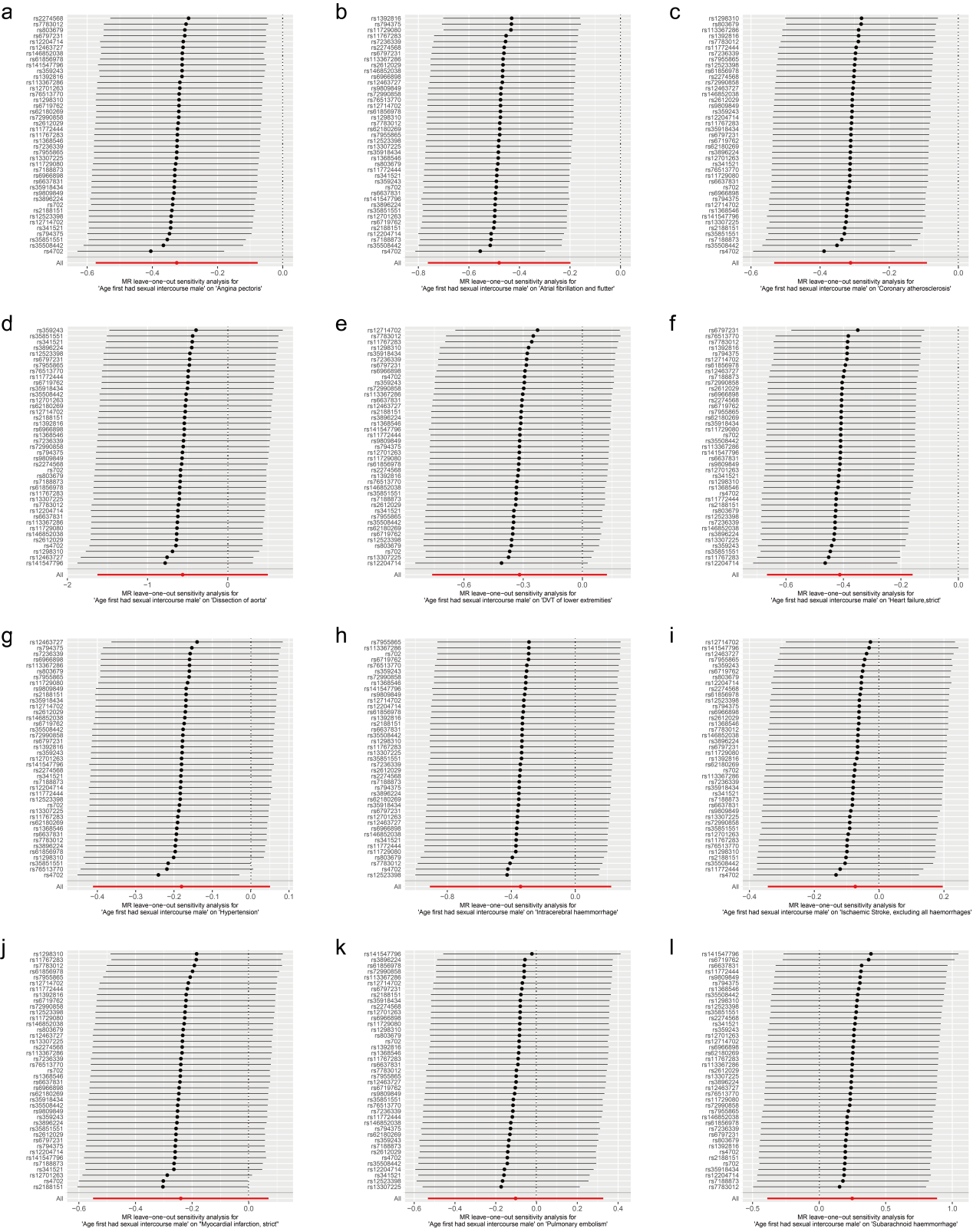


Figure S12. Forest plot of male AFS on cardiovascular disease


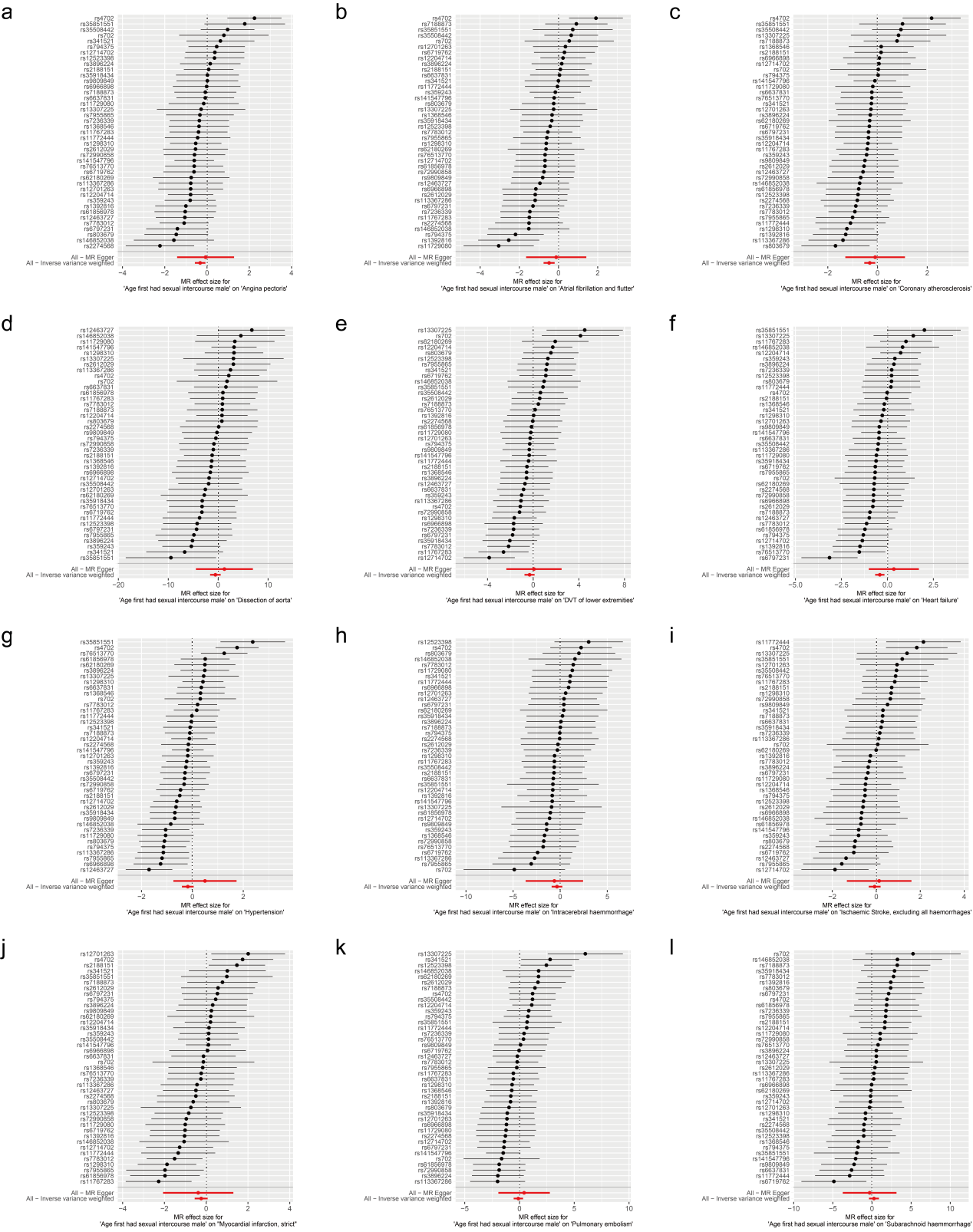


Figure S13. Scatter plot of NSP on cardiovascular disease


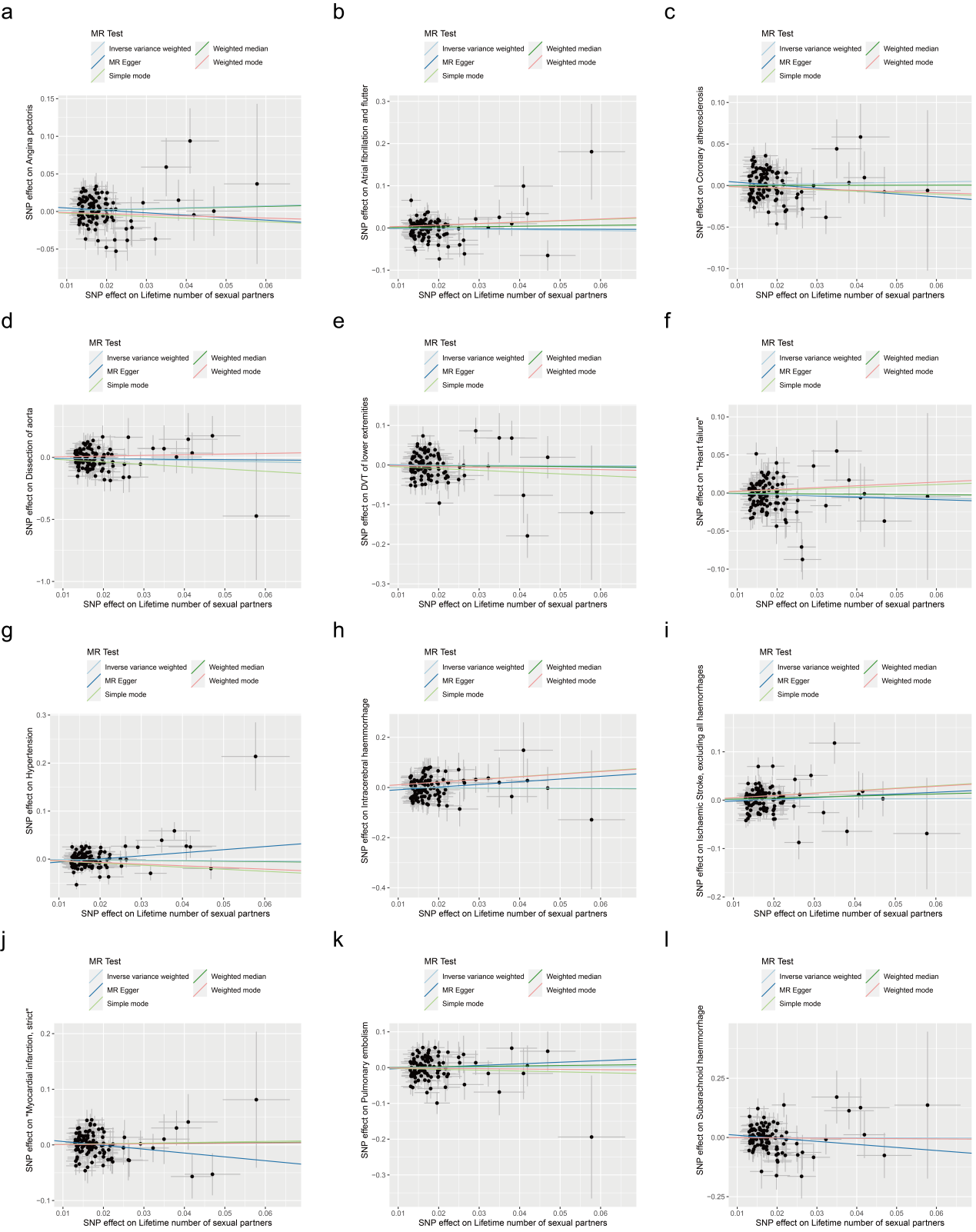


Figure S14. Funnel plot of NSP on cardiovascular disease


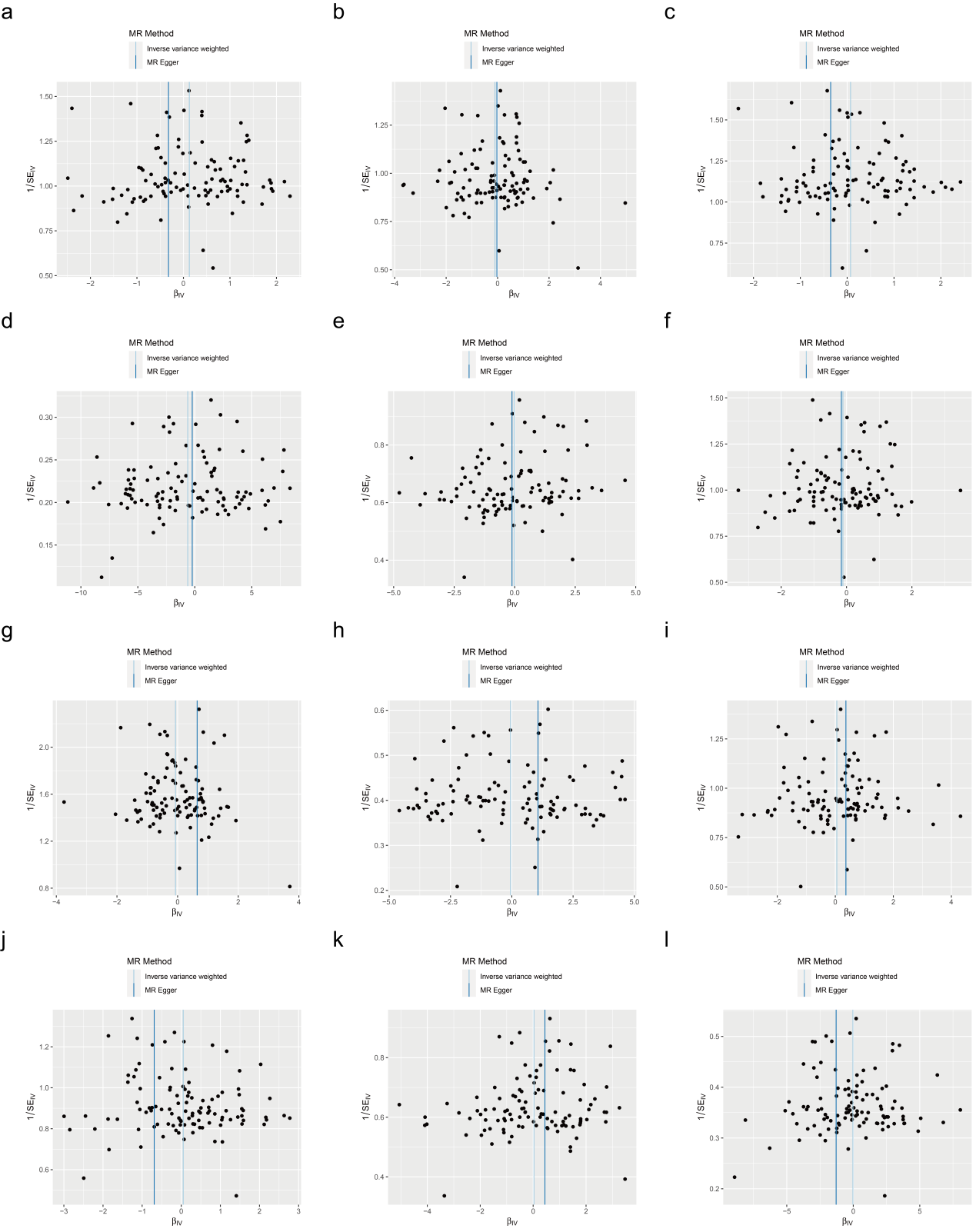


Figure S15. Leave-one-out plot of NSP on cardiovascular disease


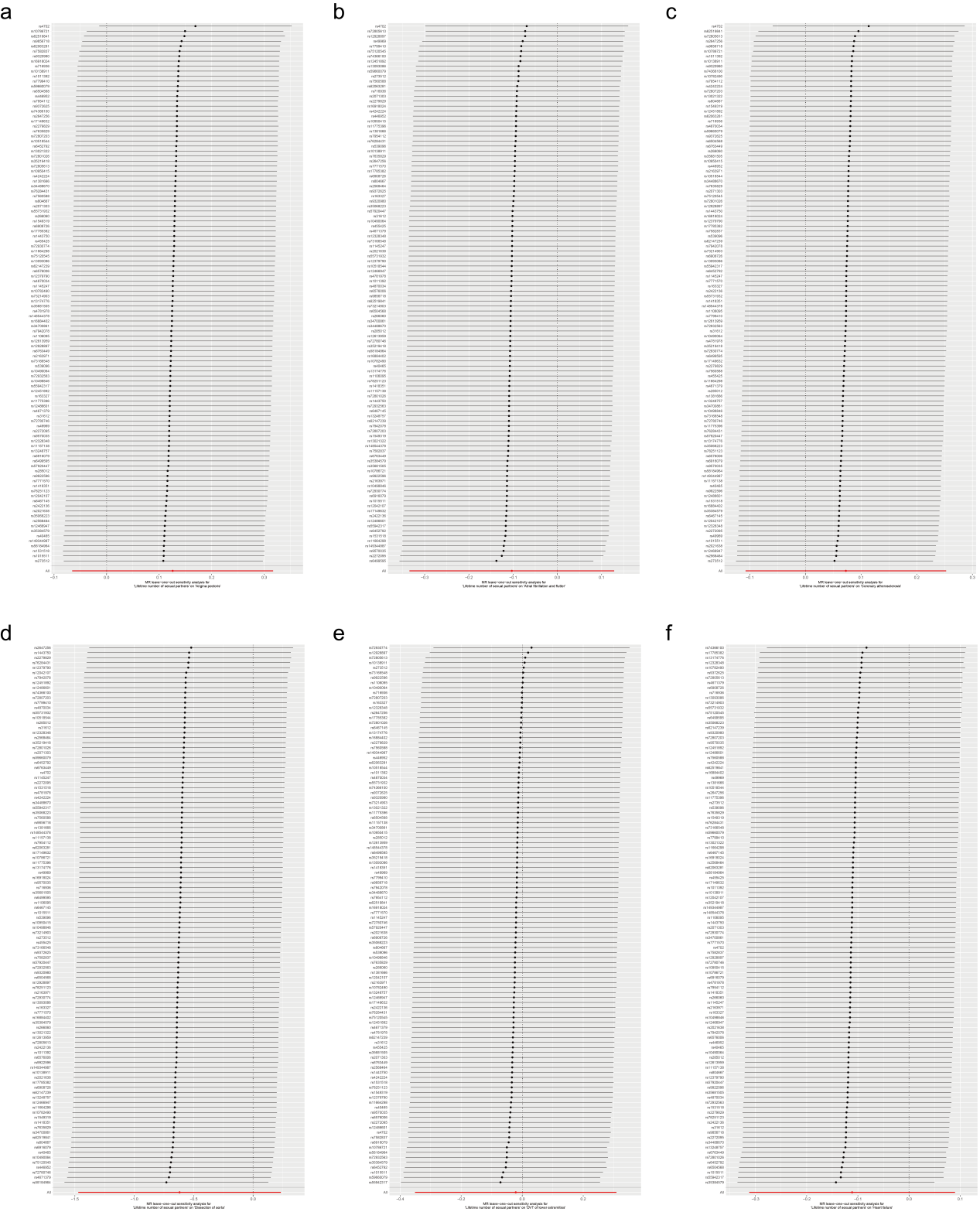


Figure S15. continued


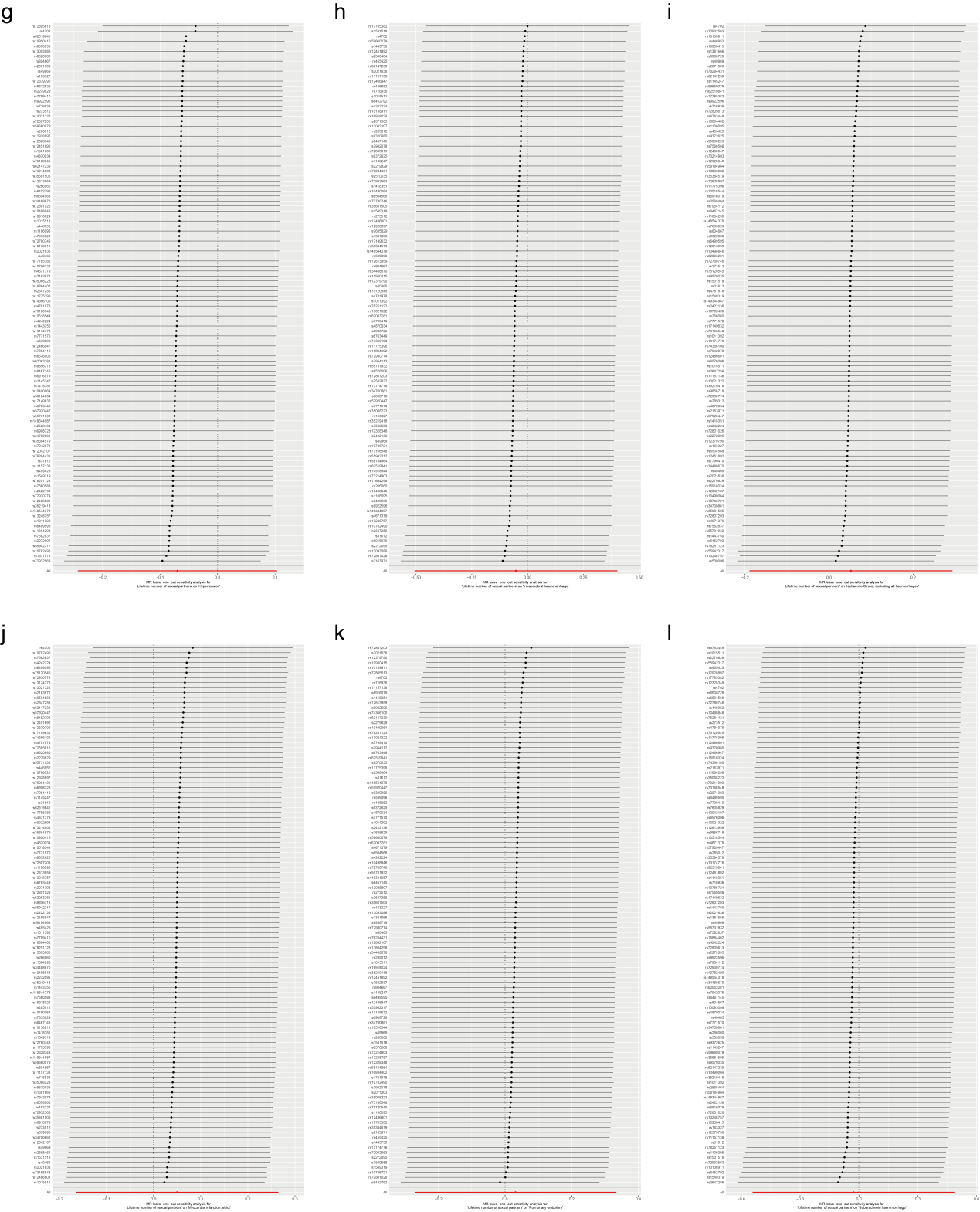


Figure S16. Forest plot of NSP on cardiovascular disease


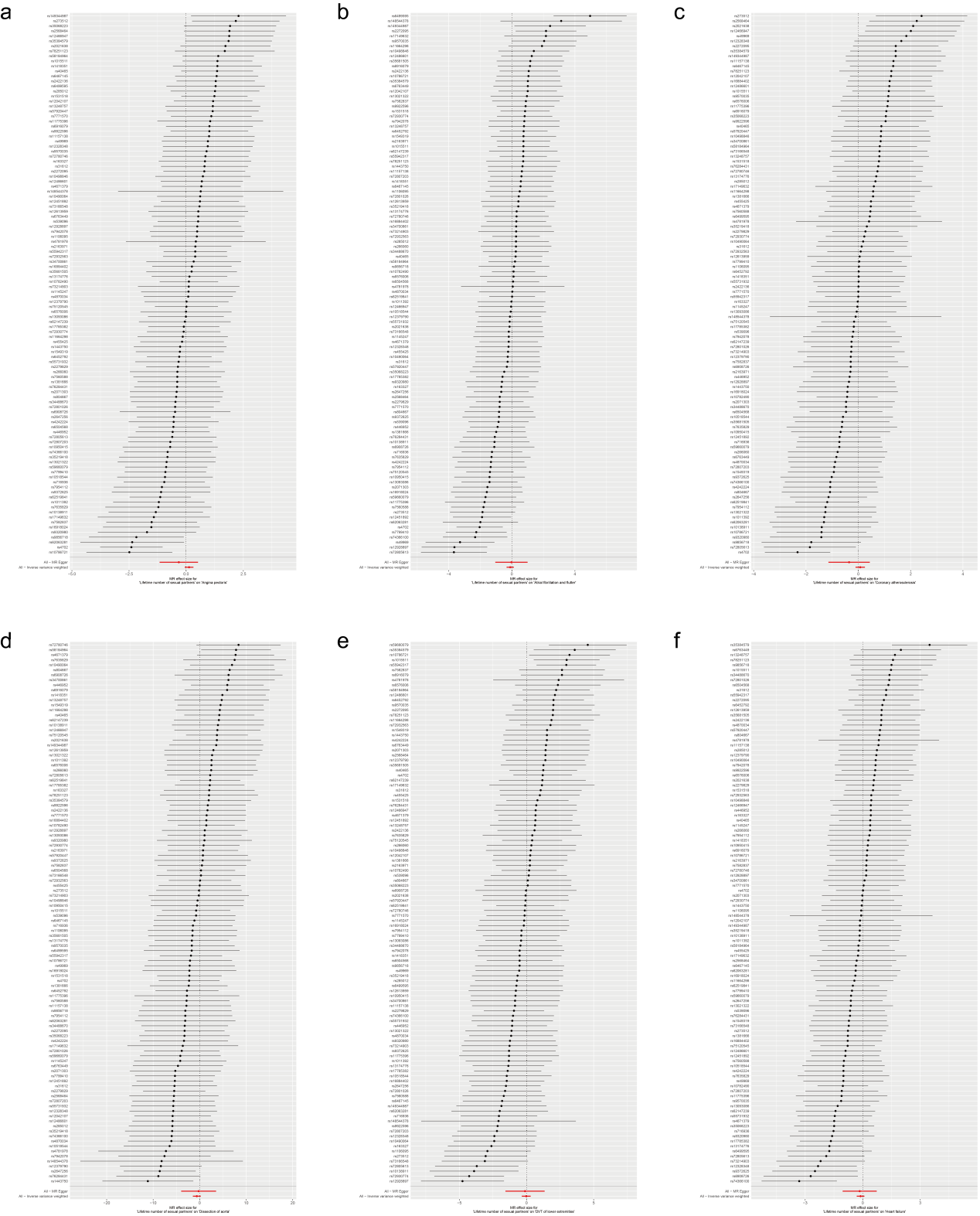


Figure S16. continued


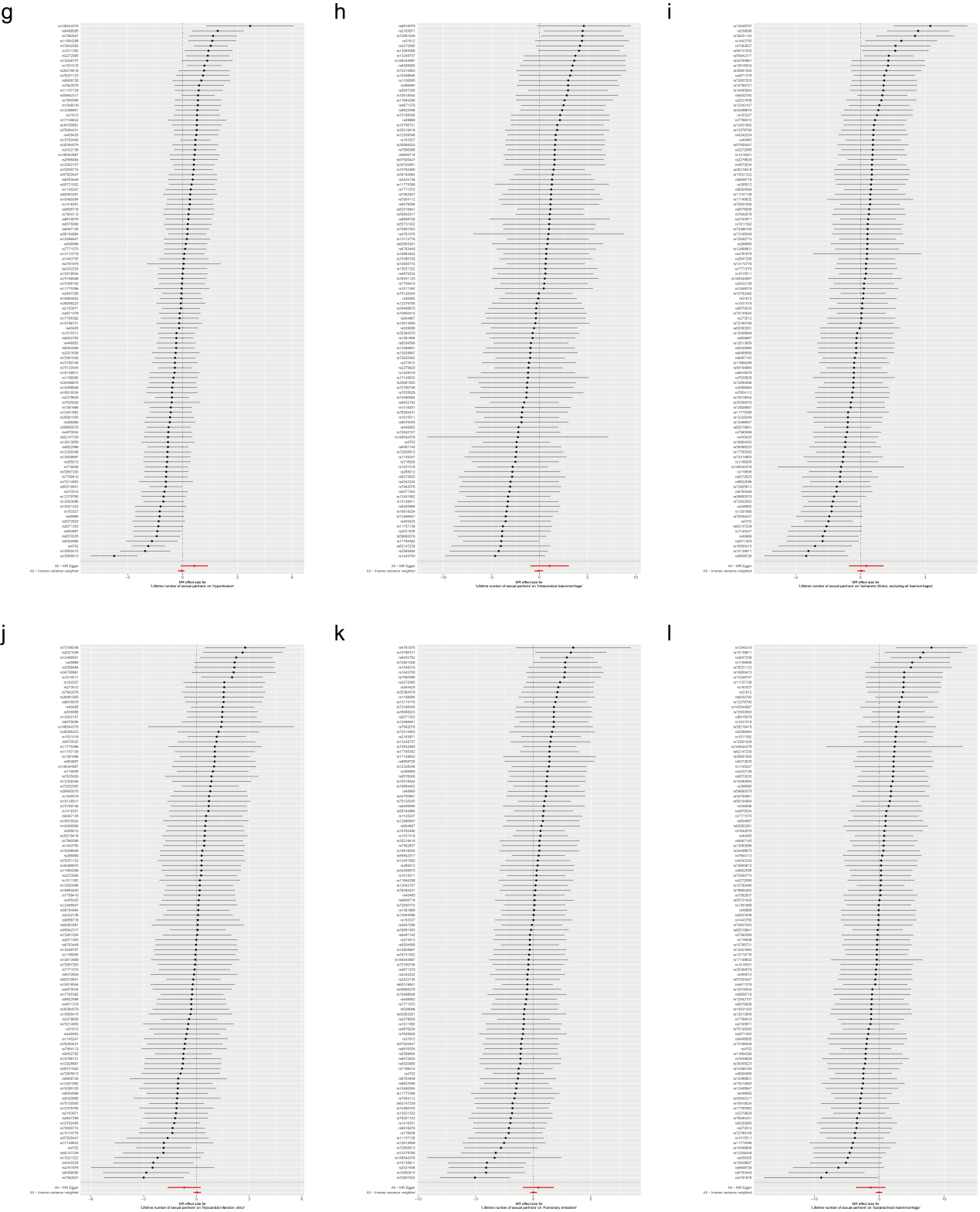


(a-l): (a) angina pectoris; (b) atrial fibrillation and flutter; (c) coronary atherosclerosis; (d) dissection of aorta; (e) DVT of lower extremities; (f) heart failure; (g) hypertension; (h) intracerebral haemmorrhage; (i) ischaemic Stroke; (j) myocardial infarction; (k) pulmonary embolism; (l) subarachnoid haemmorrhage. AFS: age at first sex; NSP: number of sexual partners.
